# Supplementary material for: Burnout and its associated factors among healthcare workers in COVID-19 isolation centres in Khartoum, Sudan: A cross-sectional study
Source: PLoS One. 2023 Jul 21;18(7):e0288638. doi: 10.1371/journal.pone.0288638 (PMC10361487; doi:10.1371/journal.pone.0288638)
Supplement: Attachment — Submitted filename: PONE-D-22-28346.pdf [file pone.0288638.s002.pdf]

PLOS ONE

Burnout and its associated factors among healthcare workers in Khartoum's COVID-19 isolation centers: A cross-sectional study

--Manuscript Draft--

**Manuscript Number:**	PONE-D-22-28346
**Article Type:**	Research Article
**Full Title:**	Burnout and its associated factors among healthcare workers in Khartoum's COVID-19 isolation centers: A cross-sectional study
**Short Title:**	Burnout and its associated factors among healthcare workers in Khartoum's COVID-19 isolation centers
**Corresponding Author:**	Elfatih A. Hasabo<br>University of Khartoum Faculty of Medicine<br>Khartoum, SUDAN
**Keywords:**	COVID-19; Burnout; healthcare workers; Sudan
**Abstract:**	Background<br><br>Burnout prevalence and its consequences on healthcare workers during the Covid-19 Pandemic are not well investigated in Sudan. This study aims to assess the prevalence of burnout and determine its associated factors among doctors and nurses during the omicron wave in Khartoum isolation centers.<br><br>Method<br><br>A multi Centre facility-based cross-sectional study was conducted between 20 February and 10 April during the last wave of COVID-19,2022. A total of 306 doctors and nurses filled out the questionnaire, with a response rate of 64.8%. They were recruited from 5 isolation centers scattered in the three localities of Khartoum. The level of burnout was assessed using an online semi-structured questionnaire based on the Oldenburg Burnout Inventory (OLBI) questionnaire. Descriptive statistics were used for continuous variables and frequencies with percentages for categorical variables. The Chi-square test and Fisher exact test were used to identify variables associated with burnout. Logistic regression was used to determine the factors associated with burnout, and the p-value of ≤ .05 is considered statistically significant.<br><br>Results<br><br>The prevalence of burnout was 45.7%. Multivariate logistic regression showed that doctors were more likely to have burnout compared with nurses (OR:1,982, CI 95% 1,2173,228 p=0,006). Also, engaged healthcare workers were less likely to suffer from burnout compared with single healthcare workers(OR: 1.982, CI 95% 1.217 – 3.228; P = 0.006). A low number of household members (p=0,017) was associated with burnout among participants.<br><br>Conclusion<br><br>There is a high prevalence of burnout among healthcare workers in Khartoum Isolation Centers, which is more apparent among doctors. Significant relations show a positive effect on burnout.
**Order of Authors:**	Elfatih A. Hasabo
	Esraa S. A. Alfadul
	Malaz Mohammed Idrees Abdalmotalib
	Salma Salah Khalid Alrawa
	Rama Osman Abdelrahman Osman
	Hadiea Mosaab AhmedElbashir Hassan

Powered by Editorial Manager® and ProduXion Manager® from Aries Systems Corporation

	Alsamany taha albasheir
	Sagad O. O. Mohamed
	Kamil Mirghani Ali Shaaban
**Additional Information:**	
**Question**	**Response**
**Financial Disclosure**<br><br>Enter a financial disclosure statement that describes the sources of funding for the work included in this submission. Review the submission guidelines for detailed requirements. View published research articles from *PLOS ONE* for specific examples.<br><br>This statement is required for submission and **will appear in the published article** if the submission is accepted. Please make sure it is accurate.<br><br>**Unfunded studies**<br>Enter: *The author(s) received no specific funding for this work.*<br><br>**Funded studies**<br>Enter a statement with the following details:<br>• Initials of the authors who received each award<br>• Grant numbers awarded to each author<br>• The full name of each funder<br>• URL of each funder website<br>• Did the sponsors or funders play any role in the study design, data collection and analysis, decision to publish, or preparation of the manuscript?<br>• **NO** - Include this sentence at the end of your statement: *The funders had no role in study design, data collection and analysis, decision to publish, or preparation of the manuscript.*<br>• **YES** - Specify the role(s) played.<br><br>* typeset	The author(s) received no specific funding for this work.
**Competing Interests**<br><br>Use the instructions below to enter a competing interest statement for this submission. On behalf of all authors, disclose any competing interests that could be perceived to bias this	The authors have declared that no competing interests exist.

work—acknowledging all financial support and any other relevant financial or non-financial competing interests.<br><br>This statement is required for submission and **will appear in the published article** if the submission is accepted. Please make sure it is accurate and that any funding sources listed in your Funding Information later in the submission form are also declared in your Financial Disclosure statement.<br><br>View published research articles from *PLOS ONE* for specific examples.<br><br>**NO authors have competing interests**<br><br>Enter: *The authors have declared that no competing interests exist.*<br><br>**Authors with competing interests**<br><br>Enter competing interest details beginning with this statement:<br><br>*I have read the journal's policy and the authors of this manuscript have the following competing interests: [insert competing interests here]*<br><br>* typeset	
**Ethics Statement**<br><br>Enter an ethics statement for this submission. This statement is required if the study involved:<br><br>• Human participants<br>• Human specimens or tissue<br>• Vertebrate animals or cephalopods<br>• Vertebrate embryos or tissues<br>• Field research<br><br>Write "N/A" if the submission does not require an ethics statement.	Ethical approval for conducting this research was obtained from the Ministry of Health - Khartoum state / Directorate General Of Curative Medicine / No: 44, Sudan. Each respondent signs a consent before participation after providing a clear explanation. Data collection was anonymized, and the confidentiality of the study participants was maintained.

Powered by Editorial Manager® and ProduXion Manager® from Aries Systems Corporation

General guidance is provided below. Consult the submission guidelines for detailed instructions. **Make sure that all information entered here is included in the Methods section of the manuscript.**<br><br>**Format for specific study types**<br><br>**Human Subject Research (involving human participants and/or tissue)**<br>• Give the name of the institutional review board or ethics committee that approved the study<br>• Include the approval number and/or a statement indicating approval of this research<br>• Indicate the form of consent obtained (written/oral) or the reason that consent was not obtained (e.g. the data were analyzed anonymously)<br><br>**Animal Research (involving vertebrate animals, embryos or tissues)**<br>• Provide the name of the Institutional Animal Care and Use Committee (IACUC) or other relevant ethics board that reviewed the study protocol, and indicate whether they approved this research or granted a formal waiver of ethical approval<br>• Include an approval number if one was obtained<br>• If the study involved *non-human primates*, add *additional details* about animal welfare and steps taken to ameliorate suffering<br>• If anesthesia, euthanasia, or any kind of animal sacrifice is part of the study, include briefly which substances and/or methods were applied<br><br>**Field Research**<br><br>Include the following details if this study involves the collection of plant, animal, or other materials from a natural setting:<br>• Field permit number<br>• Name of the institution or relevant body that granted permission	
**Data Availability**<br><br>Authors are required to make all data underlying the findings described fully	No - some restrictions will apply

available, without restriction, and from the time of publication. PLOS allows rare exceptions to address legal and ethical concerns. See the PLOS Data Policy and FAQ for detailed information.<br><br>A Data Availability Statement describing where the data can be found is required at submission. Your answers to this question constitute the Data Availability Statement and **will be published in the article**, if accepted.<br><br>**Important:** Stating 'data available on request from the author' is not sufficient. If your data are only available upon request, select 'No' for the first question and explain your exceptional situation in the text box.<br><br>Do the authors confirm that all data underlying the findings described in their manuscript are fully available without restriction?	
**Describe where the data may be found in full sentences. If you are copying our sample text, replace any instances of XXX with the appropriate details.**<br><br>• If the data are **held or will be held in a public repository**, include URLs, accession numbers or DOIs. If this information will only be available after acceptance, indicate this by ticking the box below. For example: *All XXX files are available from the XXX database (accession number(s) XXX, XXX.*).<br>• If the data are all contained **within the manuscript and/or Supporting Information files**, enter the following: *All relevant data are within the manuscript and its Supporting Information files.*<br>• If neither of these applies but you are able to provide **details of access elsewhere**, with or without limitations, please do so. For example:<br><br>*Data cannot be shared publicly because of [XXX]. Data are available from the XXX Institutional Data Access / Ethics*	The dataset generated during the current study is available from the corresponding author upon reasonable request.

Committee (contact via XXX) for researchers who meet the criteria for access to confidential data.<br><br>*The data underlying the results presented in the study are available from (include the name of the third party and contact information or URL).*<br>• This text is appropriate if the data are owned by a third party and authors do not have permission to share the data.<br><br>* typeset	
Additional data availability information:	

Powered by Editorial Manager® and ProduXion Manager® from Aries Systems Corporation

Manuscript Click here to access/download;Manuscript;manuscript.docx

Burnout and its associated factors among healthcare workers in Khartoum's COVID-19 isolation centers: A cross-sectional study

Authors: Esraa S. A. Alfadul*[1], Malaz Mohammed Idrees Abdalmotalib[1], Salma Salah Khalid Alrawa[1], Rama Osman Abdelrahman Osman[1], Hadiea Mosaab AhmedElbashir Hassan[1], Alsamany taha albasheir[1], Elfatih A. Hasabo[1], Sagad O. O. Mohamed[1], Kamil Mirghani Ali Shaaban[2].

Affiliations

1. Faculty of Medicine, University of Khartoum, Khartoum, Sudan
2. Department of Community Medicine, Faculty of Medicine, University of Khartoum, Khartoum, Sudan

*__Corresponding Author__: Esraa S. A. Alfadul, Faculty of Medicine, University of Khartoum. Al Qasr Avenue, Khartoum, Khartoum state, Sudan, 11111.

E-mail: Esraaalfadul.uofk@gmail.com

ORCID: 0000-0001-9812-5095

Abstract word count: 258

Number of tables: 4

Abstract

Background: Burnout prevalence and its consequences on healthcare workers during the Covid-19 Pandemic are not well investigated in Sudan. This study aims to assess the prevalence of burnout and determine its associated factors among doctors and nurses during the omicron wave in Khartoum isolation centers.

Method: A multi Centre facility-based cross-sectional study was conducted between 20 February and 10 April during the last wave of COVID-19,2022. A total of 306 doctors and nurses filled out the questionnaire, with a response rate of 64.8%. They were recruited from 5 isolation centers scattered in the three localities of Khartoum. The level of burnout was assessed using an online semi-structured questionnaire based on the Oldenburg Burnout Inventory (OLBI) questionnaire. Descriptive statistics were used for continuous variables and frequencies with percentages for categorical variables. The Chi-square test and Fisher exact test were used to identify variables associated with burnout. Logistic regression was used to determine the factors associated with burnout, and the p-value of ≤ .05 is considered statistically significant.

Results: The prevalence of burnout was 45.7%. Multivariate logistic regression showed that doctors were more likely to have burnout compared with nurses (OR:1,982, CI 95% 1,2173,228 p=0,006). Also, engaged healthcare workers were less likely to suffer from burnout compared with single healthcare workers(OR: 1.982, CI 95% 1.217 – 3.228; P = 0.006). A low number of household members (p=0,017) was associated with burnout among participants.

Conclusion: There is a high prevalence of burnout among healthcare workers in Khartoum Isolation Centers, which is more apparent among doctors. Significant relations show a positive effect on burnout.

Introduction

COVID-19 is a contagious respiratory disease with varying degrees of morbidity and mortality depending on the age and fitness of the individual.[1] The virus emerged on 31 December 2019 in Wuhan, Hubei Province, China. 2 and has declared a global health emergency on 30 January 2020 and a global pandemic on 11 March 2020 by the World Health Organization.[3,4,5] Sudan reported the first COVID-19 case on 13th March 2020; since the declaration of the first case, the Sudan Federal Ministry of Health has enhanced the measures to combat the spread of the virus. On 13 April, after the registration of 10 cases of COVID-19, the authorities announced a total lockdown in the state of Khartoum - the most populated state in Sudan -, suspending all gatherings, even prayers in mosques as the virus has spread beyond Khartoum, reaching other states. On 25 May, five isolation centers were established in Khartoum to isolate and deal with COVID-19 cases. Later on, the number of isolation centers increased in proportion to the high attack rate of the virus that reaching 14,401 cases with 1,116 deaths by 11 November 2020, reflecting a high fatality rate of 7.7%**.** By 7 July 2022, there were 62,745 confirmed cases of COVID-19 with 4,952 deaths in Sudan.[6-9]

Although public health measures, such as lockdowns, and social distancing, are crucial to reducing the spread of COVID-19, they increase stress, anxiety, and mental disorders.[10] The World Health Organization has recognized "burnout" as an "occupational phenomenon ."As lockdowns have significantly affected our work-life balance and work environments, much research has ascertained burnout and its contributing factors.[11]

Covid-19 has a critical psychological impact on the community.[12] The length of the pandemic period- more than two years -, the shortage of data about the virus treatment, and the unknown destiny of the Pandemic resulted in many studies that showed a significant increase in cases of anxiety, psychological stress, and depressive disorders worldwide, and because the medical staff worked under severe psychological pressure: being highly stressed by losing patients and colleagues, preference of having long shifts to protect their families, having no clear curing strategies, and urgency in instructing interpretation, all incredibly highlight peaking of burnout among healthcare providers.[13-16]

Burnout is a critical issue that generates inefficiency in healthcare organizations.[13] It lowers the quality of healthcare systems and negatively impacts patient prognosis.[17,18] Burnout affects the psychological well-being of the staff leading to medical errors,[19,20] makes the health system waste a lot of money and resources- because some workers leave their jobs so compelling the system to recruit new staff and offer a new training program and it impedes the process of psychological support for patients, which is part of the treatment.[17,19,21] Health care providers who are emotionally exhausted -express burnout- are unable to support patients neither psychologically nor fateful medical decisions.

While the Pandemic affects all the world, studies show that developed countries are affected less than developing ones. Africa is the most vulnerable region for the impact of higher mortality and morbidity, especially among healthcare providers.[16]

Sudan's revolution, the terrible economic status, collapsing health system, end-stage arrival of cases due to the centralized isolation centers, difficulty of transportation, and stigmatization of COVID-19 cases all affect the mental well-being of health care providers. Although, at present, no data considering the burnout among healthcare providers in Sudan, many published papers confirmed the high prevalence of burnout -among various countries in the Pandemic due to diversified factors - leaving no doubt that the crisis is more exacerbated in Sudan.[22,23,24]

Burnout prevalence and its consequences on healthcare workers are not well investigated in Sudan, but stigmatization of mental problems in Sudan and professional stigma of mental illness propose that healthcare providers repress their mental suffering, navigating to more emotional stress and susceptibility to burnout.[25] Sudan's health system has been

facing critical challenges, including a lack of personal protective equipment and other necessary medical equipment. As a result, healthcare workers must deal with COVID-19 without the basic equipment they need to protect the patients and themselves from the infection. Consequently, this puts a tremendous amount of pressure on the doctors, largely due to the poor contamination protection and frustration over not being able to deliver the best possible patient care.[26]

The extent of burnout, its distribution between workers in different Isolation centers, and its predictors and protective factors are essential for decision-makers. In our study, we will measure the prevalence of burnout among health care providers exposed to COVID-19 patients, so we can contribute with data helping decision-makers fight the battle of the COVID-19 Pandemic and the hidden Pandemic -burnout -.

Methods

Study design and settings

This facility-based cross-sectional study was conducted between 20 February and 10 April during the last wave of COVID-19,2022.

There are ten isolation centers in Khartoum state distributed between the Sudanese Metropolis. These centers are Ibrahim Malik Teaching Hospital, Haj El-Mardi Hospital, Al-Shaab Teaching Hospital, Omdurman, Alnaw Hospital, Ahmed Gasim Teaching Hospital, Bahri Teaching Hospital, Jabra Hospital for emergency and injuries, Omdurman, and Khartoum Teaching Hospital.

We selected the largest and smallest isolation center from each of the three cities of the Sudanese metropolis. During the last wave, Ahmed Gasim Hospital was out of service, and there was one active isolation center in the city of Bahri. Therefore, the centers finally included in this study were Alnaw Hospital and Omdurman hospital from Omdurman, Al-Shaab Teaching Hospital, and Jabra Hospital for emergency and injuries from Khartoum, beside Bahri Teaching Hospital from Bahri.

Participants

The study participants were all doctors and nurses who worked in the selected centers during the study period. No one was excluded. We contacted Collaborators from each facility, and online surveys were supplied to them to disseminate to healthcare practitioners. The total number of the targeted healthcare practitioners was 472, and 306 completed the questionnaire, yielding a response rate of 64.8%

Data collection and tool

The burnout level of the participants was assessed using an online self-administered questionnaire. The questionnaire was divided into five sections, the first of which dealt with socio-demographic characteristics such as age, gender, residence, marital status, number of household members, presence of older family members in the household, history of comorbidities, and history of mental illness.

The second part was about the professional characteristics (isolation center, job title, years of experience, working in ICU, working hours and extra duties, and experience with COVID-19 infection); the third was related to predictors of burnout (COVID-19 among relatives, colleagues, and the patient himself, fear of infection, satisfaction with protective measures of the hospital, feeling of despair about COVID 19 patients). We also investigated adaptive behaviors among the participants (exercise, spirituality, smoking, professional support, self-care, mental breaks, and procrastination), and this was the fourth part of the questionnaire.

The fifth and final part assessed burnout and was based on the Oldenburg Burnout Inventory (OLBI) questionnaire. [27] It is a 16-item validated tool for the investigation of burnout. Items consist of positively and negatively worded questions related to exhaustion and disengagement that are recorded on a four-point Likert scale (from 1 = Strongly Agree to 4 = Strongly Disagree); one point designated the lowest burnout and four designated the highest. Disengagement refers to distancing oneself from the content of one's work, while exhaustion refers to fatigue, loss of energy, feelings of emptiness, and a strong need for rest. [28,29,30] Participants were considered at high risk of burnout if they met the thresholds of 2.1 or more for the exhaustion subscales and 2.25 or more for the disengagement subscales with a cut-off point of 35. [30]

Statistical analysis plan

Data were extracted in an excel sheet, cleaned, and imported into the R software version 4.0.2 and SPSS version 28 (SPSS Inc., Chicago, IL, USA). The normality of distribution was tested using Kolmogorov- Smirnov test. Descriptive statistics were used in mean and Standard deviation for the continuous variables and

frequencies with percentages for categorical variables. The Chi-square test and Fisher exact test were used to identify variables associated with burnout. Multiple logistic regression analyses evaluated the variables that showed a statistically significant relationship at the bivariate analysis level. The p-value of $\leq .05$ was set as the significance level of the study.

Results

Participants information

A total of 306 healthcare workers, with an overall mean age of 26.7 ± 3.8, participated in the study. Nearly half of the participants were males (n=156, 51.0%), and the majority were living in Omdurman (45.1%), followed by Khartoum (42.8%). Also, most of them were singles (83.3%), nurses (58.5%), and only 30 participants (9.8%) were suffering from comorbidities. Regarding the status of COVID-19 in their relatives, 38.6% stated that their relatives had been infected with COVID-19, while 17.0% were infected and then died**. (Table 1).**

Most respondents were from Jabra Isolation Center (38.6%) and Omdurman Teaching Hospital (33.3%). Among all participants, 22.5% tested positive for COVID-19, and nearly (53.6%) were working in the ICU. (**Table 1).**

Burnouts among participants

Responses for every single item of the Oldenburg Burnout Inventory (OLBI) are shown in **(Table 2).** Burnout prevalence was 45.7% among study participants. A low number of household members (p= 0.017) and being a doctor (p= 0.003) were associated with burnout among participants. Also, marital status was significantly associated with burnout (p = 0.001)**(Table 1).** Not surprisingly, fear of infection (p < 0.001) and satisfaction with hospital safety measures (p = 0.002) were associated with burnout.

The most frequent adaptive behaviors were taking brief mental breaks throughout the day (42.2%) and exercising (37.6%) (**Table 3).** Interestingly the following factors were not associated with burnout: average income (p >0.9), working years (p = 0.8), working hours (0.8), extra hours (p = 0.065), previous working experience in COVID19 centers (p = 0.6) working center (p = 0.8) and site (p= 0.6) and fear of patient death despite all measure (p > 0.9)

The multivariate logistic regression showed doctors were more likely to have burnout than nurses (OR: 1.982, CI 95% 1.217 – 3.228; p = 0.006). Also, engaged healthcare workers were less likely to suffer burnout than single healthcare workers (OR: 3.974, CI 95% 0.143– 0.980; P = 0.045) (**Table 4).**

Discussion

The immense burden of the COVID-19 Pandemic has led to increased psychological distress among healthcare workers, especially frontline staff combating the COVID-19 Pandemic. The current study addresses a significant issue and provides a better understanding of the problem in a low-income country. In this cross-sectional study, we found that near half of the healthcare workers in the included COVID-19 isolation centers in Khartoum have suffered from burnout, in terms of emotional exhaustion and lack of personal accomplishment and engagement, a state that might have significant consequences for the healthcare providers, the patients, and the healthcare system, and represents a threat to the delivery of safe and effective healthcare.

This finding is in line with the few studies that assessed the risk of burnout among healthcare workers in Sudan, adding to the growing body of evidence suggesting a high burnout rate among hospital healthcare workers during COVID-19. One of those previous studies assessed 181 resident physicians in Sudan in 2021 through the OLBI tool and found that 71% of the participants met the criteria for burnout [32]. Another study used the Maslach Burnout Inventory assessment tool among 300 resident physicians at the teaching hospitals of Gezira state, Sudan, in 2021 and found that 86.1% of them had burnout [33]. Also, the findings of the current study are consistent with and reaffirm findings from studies done in other countries, which reported high rates of burnout among healthcare workers [34-38].

The findings of the current study and the previous studies can be explained by the psychologically demanding nature of the profession and exposure to a high level of socio-economic pressure and work-related stress, which render healthcare workers vulnerable to burnout [39]. Moreover, other factors may contribute to the increased burnout among Sudanese healthcare workers during the COVID-19 Pandemic in this study. The country's weak healthcare system, economic crisis, high inflation rates, security situation, and political instability and conflicts during this period have placed more pressure on the healthcare system and professionals [40,41]. Furthermore, dealing with the heavy load of patients presenting to the fragile health system, the lack of suitable accommodations and transportation to and from hospitals during the lockdown period, concerns about a lack of treatment supplies and personal protective equipment, and the fear of becoming infected and infecting their families may explain the observed high rate of burnout among participants.[40,41].

We found that burnout was generally not statistically associated with most of the demographic features in this study, reflecting the wide distribution of the problem among the participants despite such differences. According to our findings, a lower number of household members, unmarried individuals, and physician jobs were the only variables associated with burnout among the participants. Previous studies revealed inconsistent results on burnout risk factors among healthcare workers, and it is unclear whether that burnout follows a specific socio-demographic pattern [32,34,38]. However, some of these associations with socio-demographic factors could be explainable. For example, in our settings, doctors are the front-liners at higher risk of physical and verbal violence [40].

Regarding the habits used to lessen burnout symptoms, seeking professional support to cope with moral distress and grief was significantly associated with lower rates of burnout among the participants. The participants have used other habits to alleviate the burnout symptoms, such as exercising, spiritual habits, and taking brief mental breaks throughout the day. However, none of them showed a significant association with burnout rates in this study. The findings of this study acknowledge a high demand for appropriate support interventions and necessitate the search for more effective strategies to be implemented to reduce the risk of burnout among healthcare workers. A recent systematic review of several interventions to prevent and reduce burnout showed that both individual-focused and organizational approaches resulted in clinically meaningful reductions in burnout among physicians [42]. Individual-focused such as training to reinforce stress coping ability to help healthcare workers avoid or recover from the emotional exhaustion and disengagement that constitute burnout should be combined with addressing organizational

and workplace defects through good management of the workplace and environment, systems improvement, providing regular psychosocial support, and recruitment of additional working staff to unburden the extreme workload and limit the working hours [39] . Hostile working condition is a significant risk factor for stress and job dissatisfaction that lead to emotional exhaustion, depersonalization, and lack of personal accomplishment. Addressing individual-focused and organizational approaches is vital to reduce harmful effects on work efficiency, patients' outcomes, and the healthcare workers themselves [39] .

Though this study aims to address and quantify burnout among many frontline staff in the main COVID-19 isolation centers, the findings have some limitations; as this is a cross-sectional study, it will be challenging to draw causative relationships. The self-reported nature of the study could raise the possibility of recall bias. Also, the study was done in five sites which might limit results generalization for all healthcare workers in all settings in the country and could compromise representativeness. There is a need for further studies to address the practical interventions and to assess their effectiveness in improving working conditions and reducing the risk of burnout among healthcare workers.

Strengths and limitations

This is one of the few studies assessing the burnout burden among healthcare workers. The sample size of the participants, considered a low response population, is representative of generalizability to overall COVID-19 isolation centers in Khartoum, Sudan.

The findings of this study need to be considered in the context of some limitations; as this is a cross-sectional study, it will be challenging to draw causative relationships. The self-reported nature of the study could raise the possibility of recall bias. Using collaborators for the data collection increases the concern of selection bias.

Also, the study was done in five sites which might limit results generalization for all healthcare workers in all settings in the country and could compromise representativeness.

Conclusion

Our study has shown that nearly half of the healthcare workers in isolation centers in Khartoum have suffered from burnout during COVID-19. Several socio-demographic factors have contributed to the increased level of burnout, and multiple coping mechanisms have accounted for a lower level of burnout among healthcare workers. Nevertheless, COVID-19 has been ongoing for over 2 years, and the constant strain may exhaust healthcare workers' coping mechanisms. Consequently, the findings of this study address the high demand for appropriate interventions to be implemented to reduce the risk of burnout among frontline healthcare workers.

Acknowledgment:

We would like to thank Ahmed Abdalhalim Mohamed Omer, Omer Abdulmajed, Aya Zakareya Noor Hamid, ASMAA MOHAMED ABBAS, Aldoma Mohammed Adam Ismail, Elhadi elsiddig Elhadi, Mohammed AFEFE Hasan Salim, and Tagwa Badawe for their help in collecting the data and Mazin S. Haroun for his help in the research. Also, we would like to thank healthcare workers for participating in our study.

Contributors:

ESA, MMI, SSK, ROA, HMA, and ATA conceived and designed the study. EAH designed the statistical analysis plan, analyzed the data, and developed the figures and tables. ESA, MMI, SSK, ROA, HMA, ATA, EAH, and SOO reviewed and contributed to the interpretation. ESA, MMI, SSK, ROA, HMA, ATA, EAH, and SOO drafted the first version of the manuscript. KMA contribute to the study design and Supervision. All authors contributed to intellectual content during the drafting and revision of the work and approved the final version of the manuscript.

Funding:

The authors funded the research.

Data availability statement

The dataset generated during the current study is available from the corresponding author upon reasonable request.

Ethical approval

Ethical approval for conducting this research was obtained from the Ministry of Health - Khartoum state / Directorate General Of Curative Medicine / No: 44, Sudan. Each respondent signs a consent before participation after providing a clear explanation. Data collection was anonymized, and the confidentiality of the study participants was maintained.

Consent for publication:

All the authors gave their written and verbal consent for publication.

Conflict of interest:

The authors declare that they do not have any conflict of interest

Author's details :

References

1. Coronavirus disease (COVID-19) [Internet]. Who.int. [cited 2021 Dec 29]. Available from: https://www.who.int/health-topics/coronavirus
2. CDC. COVID-19 and your health [Internet]. Centers for Disease Control and Prevention. 2021 [cited 2021 Dec 29]. Available from: https://www.cdc.gov/coronavirus/2019-ncov/about/index.html
3. WHO declares public health emergency for novel Coronavirus [Internet]. Medscape. 2020 [cited 2021 Dec 29]. Available from: https://www.medscape.com/viewarticle/924596
4. Wee S-L, McNeil DG Jr, Hernández JC. W.H.o. Declares global emergency as Wuhan Coronavirus spreads. The New York Times [Internet]. 2020 30 January [cited 2021 29 December]; Available from: https://www.nytimes.com/2020/01/30/health/coronavirus-world-health-organization.html
5. Q5.U.S. to suspend most travel from Europe; N.b.a. pauses after player gets virus. The New York Times [Internet]. 2020 Mar 11 [cited 2021 Dec 29]; Available from: https://www.nytimes.com/2020/03/11/world/coronavirus-news.html
6. SUDAN: Full lockdown in Khartoum. (2020). Africa Research Bulletin: Economic, Financial and Technical Series, 57(3). https://doi.org/10.1111/j.1467-6346.2020.09437.x
7. Kunna E. Sudan: Managing COVID-19 pandemic during a time of transition. Arab Reform Initiative [Internet]. 2020 [cited 2022 Jul 10]; Available from: https://www.arab-reform.net/publication/sudan-managing-covid-19-pandemic-during-a-time-of-transition/
8. Ahmed A, Mohamed NS, El-Sadig SM, Fahal LA, Abelrahim ZB, Ahmed ES, Siddig EE. COVID-19 in Sudan. J Infect Dev Ctries. 2021 7 March;15(2):204-208. DOI: 10.3855/jidc.14520. PMID: 33690201.
9. Sudan: WHO Coronavirus disease (COVID-19) dashboard with vaccination data. (n.d.). Wh o.int. Retrieved 10 July, 2022, from https://covid19.who.int/region/emro/country/sd
10. Coping with stress [Internet]. Cdc.gov. 2021 [cited 2021 Dec 29]. Available from: https://www.cdc.gov/mentalhealth/stress-coping/cope-with-stress/index.html
11. Burnout [Internet]. Mental Health UK. 2021 [cited 2021 Dec 29]. Available from: https://mentalhealth-uk.org/burnout/
12. Brooks SK, Webster RK, Smith LE, Woodland L, Wessely S, Greenberg N, Rubin GJ. The psychological impact of quarantine and how to reduce it: a rapid review of the evidence. Lancet. 2020 Mar 14;395(10227):912-920. doi: 10.1016/S0140-6736(20)30460-8. Epub 2020 26 February. PMID: 32112714; PMCID: PMC7158942.
13. Leo CG, Sabina S, Tumolo MR, Bodini A, Ponzini G, Sabato E, Mincarone P. Burnout Among Healthcare Workers in the COVID-19 Era: A Review of the Existing Literature. Front Public Health. 2021 29 October;9:750529. doi: 10.3389/fpubh.2021.750529. PMID: 34778184; PMCID: PMC8585922.
14. The Lancet. COVID-19: protecting healthcare workers. Lancet [Internet]. 2020 [cited 2021 Dec 29];395(10228):922. Available from: https://www.thelancet.com/journals/lancet/article/PIIS0140-6736(20)30644-9/fulltext
15. Søvold LE, Naslund JA, Kousoulis AA, Saxena S, Qoronfleh MW, Grobler C, et al. Prioritizing the mental health and well-being of healthcare workers: An urgent global public health priority. Front Public Health [Internet]. 2021 [cited 2021 Dec 29];9:679397. Available from: https://pubmed.ncbi.nlm.nih.gov/34026720/
16. Afulani PA, Gyamerah AO, Nutor JJ, Laar A, Aborigo RA, Malechi H, Sterling M, Awoonor-Williams JK. Inadequate preparedness for response to COVID-19 is associated with stress and burnout among healthcare workers in Ghana. PLoS One. 2021 Apr 16;16(4):e0250294. doi: 10.1371/journal.pone.0250294. PMID: 33861808; PMCID: PMC8051822.

17. Patel RS, Bachu R, Adikey A, Malik M, Shah M. Factors related to physician burnout and its consequences: A review. Behav Sci (Basel) [Internet]. 2018;8(11):98. Available from: http://dx.doi.org/10.3390/bs8110098
18. Salyers MP, Bonfils KA, Luther L, Firmin RL, White DA, Adams EL, Rollins AL. The Relationship Between Professional Burnout and Quality and Safety in Healthcare: A Meta-Analysis. J Gen Intern Med. 2017 Apr;32(4):475-482. doi: 10.1007/s11606-016-3886-9. Epub 2016 26 October. PMID: 27785668; PMCID: PMC5377877.
19. Moss M, Good VS, Gozal D, Kleinpell R, Sessler CN. An Official Critical Care Societies Collaborative Statement: Burnout Syndrome in Critical Care Health Care Professionals: A Call for Action. Am J Crit Care. 2016 Jul;25(4):368-76. doi: 10.4037/ajcc2016133. PMID: 27369038.
20. Brown, S.D.; Goske, M.J.; Johnson, C.M. Beyond substance abuse: stress, burnout, and depression as causes of physician impairment and disruptive behavior. J. Am. Coll. Radiol. 2009, 6, 479–485. [CrossRef] [PubMed]
21. Pélissier C, Charbotel B, Fassier JB, Fort E, Fontana L. Nurses' Occupational and Medical Risks Factors of Leaving the Profession in Nursing Homes. Int J Environ Res Public Health. 2018 Aug 27;15(9):1850. doi: 10.3390/ijerph15091850. PMID: 30150568; PMCID: PMC6163261.
22. Lin YY, Pan YA, Hsieh YL, Hsieh MH, Chuang YS, Hsu HY, Huang YH, Hsu CE, Cheng YC, Cho SF, Wang CL. COVID-19 Pandemic Is Associated with an Adverse Impact on Burnout and Mood Disorder in Healthcare Professionals. Int J Environ Res Public Health. 2021 1 April;18(7):3654. doi: 10.3390/ijerph18073654. PMID: 33915733; PMCID: PMC8036532.
23. Nguyen J, Liu A, McKenney M, Liu H, Ang D, Elkbuli A. Impacts and challenges of the COVID-19 Pandemic on emergency medicine physicians in the United States. Am J Emerg Med. 2021 Oct;48:38-47. doi: 10.1016/j.ajem.2021.03.088. Epub 2021 2 April. PMID: 33836387; PMCID: PMC8016733.
24. Mattei A, Fiasca F, Mazzei M, Abbossida V, Bianchini V. Burnout among healthcare workers at L'Aquila: its prevalence and associated factors. Psychol Health Med [Internet]. 2017 [cited 2022 Jul 10];22(10):1262–70. Available from: https://pubmed.ncbi.nlm.nih.gov/28503931/
25. Brower KJ. Professional Stigma of Mental Health Issues: Physicians Are Both the Cause and Solution. Acad Med. 2021 1 May;96(5):635-640. doi: 10.1097/ACM.0000000000003998. PMID: 33885412; PMCID: PMC8078109.
26. El-Sadig SM, Fahal LA, Abdelrahim ZB, Ahmed ES, Mohamed NS, Siddig EE. Impact of COVID-19 on doctors and healthcare providers during the Pandemic in Sudan. Trans R Soc Trop Med Hyg. 2021 Jun 2;115(6):577-578. doi: 10.1093/trstmh/trab016. PMID: 33547896; PMCID: PMC7928645.
27. Oldenburg Burnout Inventory (OLBI). (n.d.). MDApp. Retrieved 2 June, 2022, from https://www.mdapp.co/oldenburg-burnout-inventory-olbi-calculator-606/
28. Gruszczynska E, Basinska BA, Schaufeli WB. Within- and between-person factor structure of the Oldenburg Burnout Inventory: Analysis of a diary study using multilevel confirmatory factor analysis. PLoS One. 2021 May 14;16(5):e0251257. doi: 10.1371/journal.pone.0251257.
29. Tipa RO, Tudose C, Pucarea VL. Measuring Burnout Among Psychiatric Residents Using the Oldenburg Burnout Inventory (OLBI) Instrument. J Med Life. 2019 Oct-Dec;12(4):354-360. doi: 10.25122/jml-2019-0089.
30. Summers, R. F., Gorrindo, T., Hwang, S., Aggarwal, R., & Guille, C. (2020). Well-being, burnout, and depression among North American psychiatrists: The state of our profession. The American Journal of Psychiatry, 177(10), 955–964. https://doi.org/10.1176/appi.ajp.2020.19090901
31. Maslach, C., & Leiter, M. P. (2016). Understanding the burnout experience: recent research and its implications for psychiatry. World Psychiatry: Official Journal of the World Psychiatric Association (WPA), 15(2), 103–111. https://doi.org/10.1002/wps.20311

32. El Dabbah NA, Elhadi YAM. Burnout among frontline health professionals in two African countries: a cross-sectional study from Egypt and Sudan. 2021. doi: 10.21203/RS.3.RS-951355/V1
33. Elhadi YAM, Ahmed A, Salih EB, Abdelhamed OS, Ahmed MHH, El Dabbah NA. A cross-sectional survey of burnout in a sample of resident physicians in Sudan. PLoS One. 2022. 4;17(3):e0265098. doi: 10.1371/journal.pone.0265098.
34. Denning M, Goh ET, Tan B, Kanneganti A, Almonte M, Scott A, Martin G, Clarke J, Sounderajah V, et al. Determinants of burnout and other aspects of psychological well-being in healthcare workers during the Covid-19 Pandemic: A multinational cross-sectional study. PLoS One. 2021 Apr 16;16(4):e0238666. doi: 10.1371/journal.pone.0238666.
35. Elhadi M, Msherghi A, Elgzairi M, Alhashimi A, Bouhuwaish A, Biala M, Abuelmeda S, Khel S, Khaled A, Alsoufi A, Elmabrouk A, Alshiteewi FB, Hamed TB, Alhadi B, Alhaddad S, Elhadi A, Zaid A. Burnout Syndrome Among Hospital Healthcare Workers During the COVID-19 Pandemic and Civil War: A Cross-Sectional Study. Front Psychiatry. 2020 Dec 11;11:579563. doi: 10.3389/fpsyt.2020.579563.
36. Chou L.-P., Li C.-Y., Hu S.C. Job stress and burnout in hospital employees: Comparisons of different medical professions in a regional hospital in Taiwan. BMJ Open. 2014;4:e004185
37. Sarboozi Hoseinabadi T, Kakhki S, Teimori G, Nayyeri S. Burnout and its influencing factors between frontline nurses and nurses from other wards during the outbreak of Coronavirus Disease -COVID-19- in Iran. Invest Educ Enferm. 2020 Jul;38(2):e3. DOI: 10.17533/udea.iee.v38n2e03.
38. Tan BYQ, Kanneganti A, Lim LJH, Tan M, Chua YX, Tan L, Sia CH, Denning M, Goh ET, Purkayastha S, Kinross J, Sim K, Chan YH, Ooi SBS. Burnout and Associated Factors Among Health Care Workers in Singapore During the COVID-19 Pandemic. J Am Med Dir Assoc. 2020 Dec;21(12):1751-1758.e5. doi: 10.1016/j.jamda.2020.09.035.
39. Elhadi M, Msherghi A, Elgzairi M, Alhashimi A, Bouhuwaish A, Biala M, Abuelmeda S, Khel S, Khaled A, Alsoufi A, Elhadi A, BenGhatnsh A. The Mental Well-Being of Frontline Physicians Working in Civil Wars Under Coronavirus Disease 2019 Pandemic Conditions. Front Psychiatry. 2021 Jan 14;11:598720. doi: 10.3389/fpsyt.2020.598720.
40. Elhadi YAM, Adebisi YA, Hassan KF, Mohammed SEE, Lin X, Lucero-Prisno Iii DE. The formidable task of fighting COVID-19 in Sudan. Pan Afr Med J. 2020 7 August;35(Suppl 2):137. doi: 10.11604/pamj.supp.2020.35.137.24984.
41. El-Sadig SM, Fahal LA, Abdelrahim ZB, Ahmed ES, Mohamed NS, Siddig EE. Impact of COVID-19 on doctors and healthcare providers during the Pandemic in Sudan. Trans R Soc Trop Med Hyg. 2021 Jun 2;115(6):577-578. doi: 10.1093/trstmh/trab016.
42. West, Colin P., et al. “Interventions to Prevent and Reduce Physician Burnout: A Systematic Review and Meta-Analysis.” Lancet, vol. 388, no. 10057, 2016, pp. 2272–2281, doi:10.1016/S0140-6736(16)31279-X.

Table 1: Baseline characteristics and demographic data of included participants.

Variables	N	Overall, N = 306[1]	Burnout No, N = 166[1]	Burnout Yes, N = 140[1]	p-value[2]
**Age**	306	26.7 ± 3.8	26.8 ± 3.7	26.6 ± 4.0	0.7
**Gender**	306				0.8
Female		150 (49.0%)	80 (48.2%)	70 (50.0%)	
Male		156 (51.0%)	86 (51.8%)	70 (50.0%)	
**Residency**	306				0.9
Bahari		37 (12.1%)	19 (11.4%)	18 (12.9%)	
Khartoum		131 (42.8%)	70 (42.2%)	61 (43.6%)	
Omdurman		138 (45.1%)	77 (46.4%)	61 (43.6%)	
**Marital status**	306				**0.001**
Engaged		26 (8.5%)	20 (12.0%)	6 (4.3%)	
Married		22 (7.2%)	5 (3.0%)	17 (12.1%)	
Single		255 (83.3%)	140 (84.3%)	115 (82.1%)	
Widowed		3 (1.0%)	1 (0.6%)	2 (1.4%)	
**Number of household members**	299	6.4 ± 2.9	6.8 ± 2.5	6.0 ± 3.2	**0.017**
**Do you live with an elderly household member who has a chronic disease?**	306	131 (42.8%)	76 (45.8%)	55 (39.3%)	0.3
**Do you suffer from any comorbidities?**	306	30 (9.8%)	14 (8.4%)	16 (11.4%)	0.4
**Do you have a history of mental illness?**	306	7 (2.3%)	2 (1.2%)	5 (3.6%)	0.3
**Do you have relative/s who have been infected/died from Covid-19?**	306				0.7
Infected		118 (38.6%)	64 (38.6%)	54 (38.6%)	
Infected and died		52 (17.0%)	31 (18.7%)	21 (15.0%)	
None		136 (44.4%)	71 (42.8%)	65 (46.4%)	
**Do you have colleague/s who have been infected/died from Covid-19?**	306				0.8
Infected		230 (75.2%)	127 (76.5%)	103 (73.6%)	
Infected and died		17 (5.6%)	8 (4.8%)	9 (6.4%)	
None		59 (19.3%)	31 (18.7%)	28 (20.0%)	
**What was your COVID-19 Screening test result?**	306				0.088
Not Tested		131 (42.8%)	72 (43.4%)	59 (42.1%)	
Tested negative		106 (34.6%)	64 (38.6%)	42 (30.0%)	
Tested positive		69 (22.5%)	30 (18.1%)	39 (27.9%)	
**What is your Job title?**	306				**0.003**
Doctor		127 (41.5%)	56 (33.7%)	71 (50.7%)	
Nurse		179 (58.5%)	110 (66.3%)	69 (49.3%)	
**What is your perceived average income?**	306				>0.9
High		5 (1.6%)	3 (1.8%)	2 (1.4%)	
Low		116 (37.9%)	64 (38.6%)	52 (37.1%)	
Medium		185 (60.5%)	99 (59.6%)	86 (61.4%)	
**How many years have you been working in this job?**	306				0.8
0		2 (0.7%)	2 (1.2%)	0 (0.0%)	
0.5		2 (0.7%)	0 (0.0%)	2 (1.4%)	
1		134 (43.8%)	70 (42.2%)	64 (45.7%)	
1.5		4 (1.3%)	1 (0.6%)	3 (2.1%)	
12		2 (0.7%)	1 (0.6%)	1 (0.7%)	
14		1 (0.3%)	0 (0.0%)	1 (0.7%)	
15		1 (0.3%)	1 (0.6%)	0 (0.0%)	
17		1 (0.3%)	1 (0.6%)	0 (0.0%)	
18		1 (0.3%)	1 (0.6%)	0 (0.0%)	
2		76 (24.8%)	38 (22.9%)	38 (27.1%)	
20		1 (0.3%)	1 (0.6%)	0 (0.0%)	
27		1 (0.3%)	1 (0.6%)	0 (0.0%)	
3		37 (12.1%)	24 (14.5%)	13 (9.3%)	
4		14 (4.6%)	8 (4.8%)	6 (4.3%)	
5		15 (4.9%)	9 (5.4%)	6 (4.3%)	
54		1 (0.3%)	1 (0.6%)	0 (0.0%)	
6		9 (2.9%)	5 (3.0%)	4 (2.9%)	
7		4 (1.3%)	2 (1.2%)	2 (1.4%)	
**In which isolation center do you work?**	306				0.8
AlNaw Teaching Hospital		19 (6.2%)	12 (7.2%)	7 (5.0%)	
Al-Shaab Hospital		44 (14.4%)	21 (12.7%)	23 (16.4%)	
Bahri Hospital		23 (7.5%)	12 (7.2%)	11 (7.9%)	

Jebra Isolation center		118 (38.6%)	65 (39.2%)	53 (37.9%)	
Omdurman Teaching Hospital		102 (33.3%)	56 (33.7%)	46 (32.9%)	
**What was your way of employment?**	306				>0.9
Appointed by the Ministry of Health		49 (16.0%)	26 (15.7%)	23 (16.4%)	
Attachment		22 (7.2%)	13 (7.8%)	9 (6.4%)	
Compulsory service		19 (6.2%)	11 (6.6%)	8 (5.7%)	
Contracts: appointed by the hospital		216 (70.6%)	116 (69.9%)	100 (71.4%)	
**Did you work in isolation centers in the previous Covid-19 waves other than the current Omicron wave?**	306	201 (65.7%)	111 (66.9%)	90 (64.3%)	0.6
**Do you work extra duty or extra hours per week?**	306	194 (63.4%)	113 (68.1%)	81 (57.9%)	0.065
**In the isolation center, do you work in the ward or in the intensive care unit (ICU)?**	306				0.6
ICU		164 (53.6%)	91 (54.8%)	73 (52.1%)	
The ward		142 (46.4%)	75 (45.2%)	67 (47.9%)	
**Working hours per week**	306	44.2 ± 18.9	44.5 ± 18.7	43.8 ± 19.2	0.8
**I'm afraid of getting infected by Covid-19 throughout my work.**	306				**<0.001**
1		85 (27.8%)	61 (36.7%)	24 (17.1%)	
2		44 (14.4%)	29 (17.5%)	15 (10.7%)	
3		99 (32.4%)	44 (26.5%)	55 (39.3%)	
4		43 (14.1%)	19 (11.4%)	24 (17.1%)	
5		35 (11.4%)	13 (7.8%)	22 (15.7%)	
**I feel safe with the protective measures taken by the hospital.**	306				**0.002**
1		71 (23.2%)	46 (27.7%)	25 (17.9%)	
2		77 (25.2%)	44 (26.5%)	33 (23.6%)	
3		90 (29.4%)	48 (28.9%)	42 (30.0%)	
4		41 (13.4%)	23 (13.9%)	18 (12.9%)	
5		27 (8.8%)	5 (3.0%)	22 (15.7%)	
**I feel the patient would die no matter what I did.**	306				>0.9
1		17 (5.6%)	10 (6.0%)	7 (5.0%)	
2		34 (11.1%)	19 (11.4%)	15 (10.7%)	
3		66 (21.6%)	38 (22.9%)	28 (20.0%)	
4		92 (30.1%)	49 (29.5%)	43 (30.7%)	
5		97 (31.7%)	50 (30.1%)	47 (33.6%)	

[1] Mean ± SD; n (%)

[2] Two Sample t-test; Pearson's Chi-squared test; Fisher's exact test

Note: 1 = Strongly Agree; 2 = Agree; 3 = Neutral; 4 = Disagree; 5 = Strongly Disagree

Table 2: Responses of questions for burnout tool.

Variables	Overall, N = 306[1]	Burnout		p-value[2]
		**No**, N = 166[1]	**Yes**, N = 140[1]	
**I always find new and interesting aspects in my work.**				**<0.001**
1	113 (36.9%)	76 (45.8%)	37 (26.4%)	
2	118 (38.6%)	62 (37.3%)	56 (40.0%)	
3	51 (16.7%)	18 (10.8%)	33 (23.6%)	
4	24 (7.8%)	10 (6.0%)	14 (10.0%)	
**There are days when I feel tired before I arrive at work.**				**<0.001**
1	135 (44.1%)	94 (56.6%)	41 (29.3%)	
2	108 (35.3%)	56 (33.7%)	52 (37.1%)	
3	42 (13.7%)	9 (5.4%)	33 (23.6%)	
4	21 (6.9%)	7 (4.2%)	14 (10.0%)	
**It happens more and more often that I talk about my work in a negative way.**				**0.005**
1	62 (20.3%)	43 (25.9%)	19 (13.6%)	

2	99 (32.4%)	59 (35.5%)	40 (28.6%)	
3	77 (25.2%)	33 (19.9%)	44 (31.4%)	
4	68 (22.2%)	31 (18.7%)	37 (26.4%)	
**After work, I tend to need more time than in the past in order to relax and feel better.**				**<0.001**
1	137 (44.8%)	98 (59.0%)	39 (27.9%)	
2	92 (30.1%)	39 (23.5%)	53 (37.9%)	
3	52 (17.0%)	23 (13.9%)	29 (20.7%)	
4	25 (8.2%)	6 (3.6%)	19 (13.6%)	
**I can tolerate the pressure of my work very well.**				**<0.001**
1	113 (36.9%)	77 (46.4%)	36 (25.7%)	
2	121 (39.5%)	63 (38.0%)	58 (41.4%)	
3	54 (17.6%)	21 (12.7%)	33 (23.6%)	
4	18 (5.9%)	5 (3.0%)	13 (9.3%)	
**Lately, I tend to think less at work and do my job almost mechanically.**				**<0.001**
1	71 (23.2%)	57 (34.3%)	14 (10.0%)	
2	123 (40.2%)	68 (41.0%)	55 (39.3%)	
3	66 (21.6%)	22 (13.3%)	44 (31.4%)	
4	46 (15.0%)	19 (11.4%)	27 (19.3%)	
**I find my work to be a positive challenge.**				**<0.001**
1	131 (42.8%)	89 (53.6%)	42 (30.0%)	
2	109 (35.6%)	57 (34.3%)	52 (37.1%)	
3	48 (15.7%)	15 (9.0%)	33 (23.6%)	
4	18 (5.9%)	5 (3.0%)	13 (9.3%)	
**During my work, I often feel emotionally drained.**				**<0.001**
1	99 (32.4%)	72 (43.4%)	27 (19.3%)	
2	104 (34.0%)	59 (35.5%)	45 (32.1%)	
3	63 (20.6%)	18 (10.8%)	45 (32.1%)	
4	40 (13.1%)	17 (10.2%)	23 (16.4%)	
**Over time, one can become disconnected from this type of work.**				**<0.001**
1	93 (30.4%)	67 (40.4%)	26 (18.6%)	
2	110 (35.9%)	61 (36.7%)	49 (35.0%)	
3	70 (22.9%)	25 (15.1%)	45 (32.1%)	
4	33 (10.8%)	13 (7.8%)	20 (14.3%)	
**After working, I have enough energy for my leisure activities.**				0.3
1	38 (12.4%)	25 (15.1%)	13 (9.3%)	
2	79 (25.8%)	44 (26.5%)	35 (25.0%)	
3	77 (25.2%)	37 (22.3%)	40 (28.6%)	
4	112 (36.6%)	60 (36.1%)	52 (37.1%)	
**Sometimes I feel sickened by my work tasks.**				**<0.001**
1	83 (27.1%)	63 (38.0%)	20 (14.3%)	
2	109 (35.6%)	63 (38.0%)	46 (32.9%)	
3	81 (26.5%)	30 (18.1%)	51 (36.4%)	
4	33 (10.8%)	10 (6.0%)	23 (16.4%)	
**After my work, I usually feel worn out and weary.**				**<0.001**
1	133 (43.5%)	100 (60.2%)	33 (23.6%)	
2	97 (31.7%)	49 (29.5%)	48 (34.3%)	
3	56 (18.3%)	15 (9.0%)	41 (29.3%)	
4	20 (6.5%)	2 (1.2%)	18 (12.9%)	
**This is the only type of work that I can imagine myself doing.**				**<0.001**
1	57 (18.6%)	45 (27.1%)	12 (8.6%)	
2	67 (21.9%)	49 (29.5%)	18 (12.9%)	
3	106 (34.6%)	48 (28.9%)	58 (41.4%)	
4	76 (24.8%)	24 (14.5%)	52 (37.1%)	
**Usually, I can manage the amount of my work well.**				**<0.001**
1	112 (36.6%)	75 (45.2%)	37 (26.4%)	
2	140 (45.8%)	73 (44.0%)	67 (47.9%)	
3	41 (13.4%)	15 (9.0%)	26 (18.6%)	
4	13 (4.2%)	3 (1.8%)	10 (7.1%)	
**I feel more and more engaged in my work.**				**<0.001**
1	76 (24.8%)	63 (38.0%)	13 (9.3%)	
2	140 (45.8%)	79 (47.6%)	61 (43.6%)	
3	65 (21.2%)	18 (10.8%)	47 (33.6%)	
4	25 (8.2%)	6 (3.6%)	19 (13.6%)	
**When I work, I usually feel energized**				**<0.001**
1	79 (25.8%)	59 (35.5%)	20 (14.3%)	

2	131 (42.8%)	64 (38.6%)	67 (47.9%)	
3	67 (21.9%)	31 (18.7%)	36 (25.7%)	
4	29 (9.5%)	12 (7.2%)	17 (12.1%)	
**Score**	34.2 ± 5.3	30.4 ± 3.1	38.8 ± 3.5	**<0.001**

[1] n (%)

[2] Pearson's Chi-squared test; Fisher's exact test

Note: 1 = Strongly Agree; 2 = Agree; 3 = Disagree; 4 = Strongly Disagree

Table 3: Habits that participants have used.

**Variables**	**Overall**, N = 306[1]	**Burnout**		**p-value**[2]
		**No**, N = 166[1]	**Yes**, N = 140[1]	
**Exercise**	115 (37.6%)	61 (36.7%)	54 (38.6%)	0.7
**spiritual habits**	107 (35.0%)	63 (38.0%)	44 (31.4%)	0.2
**Smoking**	35 (11.4%)	18 (10.8%)	17 (12.1%)	0.7
**Seek professional support to cope with moral distress and grief**	41 (13.4%)	32 (19.3%)	9 (6.4%)	**0.001**
**Take brief mental breaks throughout the day**	129 (42.2%)	75 (45.2%)	54 (38.6%)	0.2
**Performing Self-care Routines**	108 (35.3%)	56 (33.7%)	52 (37.1%)	0.5
**Poor Time Management and Procrastination**	80 (26.1%)	49 (29.5%)	31 (22.1%)	0.14

[1] n (%)

[2] Pearson's Chi-squared test; Fisher's exact test

Table 4: Multivariate logistic regression for predictors of burnout among healthcare workers

Variables	Multivariate Logistic regression			
	OR	95% CI		p-value
		Lower	Upper	
**Marital status**				
Single	-	-	-	-
Married	3.974	1.367	11.547	0.011
Engaged	0.374	0.143	0.980	0.045
Widowed	2.289	0.199	26.317	0.506
**Number of household members**	0.948	0.867	1.036	0.241
**What is your Job title?**				
Nurse	-	-	-	-
Doctor	1.982	1.217	3.228	0.006

CI: Confidence Interval, OR= Odd ratio
