# Supplementary material for: Burnout and its associated factors among healthcare workers in COVID-19 isolation centres in Khartoum, Sudan: A cross-sectional study
Source: PLoS One. 2023 Jul 21;18(7):e0288638. doi: 10.1371/journal.pone.0288638 (PMC10361487; doi:10.1371/journal.pone.0288638)
Supplement: Attachment — Submitted filename: PONE-D-22-28346_R1.pdf [file pone.0288638.s004.pdf]

PLOS ONE

Burnout and its associated factors among healthcare workers in Khartoum's COVID-19 isolation centers: A cross-sectional study

--Manuscript Draft--

**Manuscript Number:**	PONE-D-22-28346R1
**Article Type:**	Research Article
**Full Title:**	Burnout and its associated factors among healthcare workers in Khartoum's COVID-19 isolation centers: A cross-sectional study
**Short Title:**	Burnout and its associated factors among healthcare workers in Khartoum's COVID-19 isolation centers
**Corresponding Author:**	Esraa S. A. Alfadul<br>University of Khartoum Faculty of Medicine<br>Khartoum SUDAN
**Keywords:**	COVID-19; Burnout; healthcare workers; Sudan
**Abstract:**	Background: Burnout prevalence and its consequences on healthcare workers during the Coronavirus Disease 2019 pandemic are not well investigated in Sudan. This study aims to assess the prevalence of burnout and its associated factors among doctors and nurses during the omicron wave in Khartoum isolation centers.<br>Method: A multi Centre facility-based cross-sectional study was conducted between 20th February and 10th April 2022 during the last wave of Coronavirus Disease 2019. A total of 306 doctors and nurses filled out the questionnaire, with a response rate of 64.8%. They were recruited from 5 isolation centers scattered in the three localities of Khartoum. The level of burnout was assessed using an online semi-structured questionnaire based on the Oldenburg Burnout Inventory questionnaire. Descriptive statistics were used for continuous variables and frequencies with percentages for categorical variables. The Chi-square test and Fisher exact test were used to identify variables associated with burnout. Logistic regression was used to determine the factors associated with burnout, and the p-value of ≤ .05 is considered statistically significant.<br><br>Results: The prevalence of burnout was 45.7%. Multivariate logistic regression showed doctors were more likely to have burnout than nurses (OR:1,982, CI 95% 1,2173,228 p=0,006). Also, engaged healthcare workers were less likely to suffer from burnout than single healthcare workers (OR: 1.982, CI 95% 1.217 – 3.228; p =.006). A low number of household members (p= .017) was associated with burnout among participants.<br><br>Conclusion: There is a high prevalence of burnout among healthcare workers in Khartoum Isolation Centers, which is more apparent among doctors. Significant relations show a positive effect on burnout.
**Order of Authors:**	Esraa S. A. Alfadul
	Malaz Mohammed Idrees Abdalmotalib
	Salma Salah Khalid Alrawa
	Rama Osman Abdelrahman Osman
	Hadiea Mosaab AhmedElbashir Hassan
	Alsamany taha albasheir
	Elfatih A. Hasabo
	Sagad O. O. Mohamed
	Kamil Mirghani Ali Shaaban

Powered by Editorial Manager® and ProduXion Manager® from Aries Systems Corporation

**Response to Reviewers:**	27-janouary-2023<br>Author’s response to reviews<br>Subject: Revision and resubmission of PONE-D-22-28346<br><br>Dear reviewer,<br>On behalf of all the contributing authors, I would like to express our sincere appreciation of the reviewers’ constructive comments concerning our article entitled “Burnout and its associated factors among healthcare workers in Khartoum's COVID-19 isolation centers: A cross-sectional study”. We have revised our manuscript according to the reviewers’ comments, questions, and suggestions. We believe that the manuscript has been further improved and the revised paragraphs were highlighted in yellow in the manuscript . If there are any other modifications we could make, we would very much like to modify them and we appreciate your help. Below are point-by-point responses to the reviewers' comments.<br><br>Your sincerely<br><br>Reviewer #1: The manuscript, although it gets the meaning across, is not very well written. It should be re-written to bring the standard close to that of the Journal's. The Discussion Section in particular can be improved; although studies have been included but their findings have not been connected with those of the study being discussed.<br>response to reviewer 1:<br>Thank you for your comment. The manuscript was revised and corrected, and the discussion section was improved according to your note.<br><br>Reviewer #2:<br>Comment: In abstract you should show prevalence of burnout<br>Response: done<br>Comment: -There should not be any abbreviations in the abstract.<br>Response: all abbreviations in the abstract were removed<br>Comment: -In introduction you should give a definition of job burnout<br>Response: a brief description of burnout was added (end of page 3)<br>Comment: -You should describe more literature on their association and on their prevalence in healthcare professionals during the covid 19 pandemic.<br>Response: more description was added to the introduction section (end of page 3)<br>Comment: -The discussion needs significant reworking. It does not explicate the reasons for nurses to have burnout.<br>Response: discussion section was revised and edited according to your note. We think the reviewer meant ‘doctors’ not nurses in his comment (see table 4)<br>Comment: Many bibliographies are obsolete. The bibliographic citations used are more than 5 years old. The authors must update and arrange the bibliography. Some references are incomplete or have errors. The authors should review this section<br>Response: we re-checked all references to ensure that all of them are not older than 2018
**Additional Information:**	
**Question**	**Response**
**Financial Disclosure**<br><br>Enter a financial disclosure statement that describes the sources of funding for the work included in this submission. Review the submission guidelines for detailed requirements. View published research articles from *PLOS ONE* for specific examples.<br><br>This statement is required for submission and **will appear in the published article** if the submission is accepted. Please make	The author(s) received no specific funding for this work.

sure it is accurate.<br><br>**Unfunded studies**<br>Enter: *The author(s) received no specific funding for this work.*<br><br>**Funded studies**<br>Enter a statement with the following details:<br>• Initials of the authors who received each award<br>• Grant numbers awarded to each author<br>• The full name of each funder<br>• URL of each funder website<br>• Did the sponsors or funders play any role in the study design, data collection and analysis, decision to publish, or preparation of the manuscript?<br>• **NO** - Include this sentence at the end of your statement: *The funders had no role in study design, data collection and analysis, decision to publish, or preparation of the manuscript.*<br>• **YES** - Specify the role(s) played.<br><br>* typeset	
**Competing Interests**<br><br>Use the instructions below to enter a competing interest statement for this submission. On behalf of all authors, disclose any competing interests that could be perceived to bias this work—acknowledging all financial support and any other relevant financial or non-financial competing interests.<br><br>This statement is required for submission and **will appear in the published article** if the submission is accepted. Please make sure it is accurate and that any funding sources listed in your Funding Information later in the submission form are also declared in your Financial Disclosure statement.<br><br>View published research articles from *PLOS ONE* for specific examples.	The authors have declared that no competing interests exist.

**NO authors have competing interests**<br><br>Enter: *The authors have declared that no competing interests exist.*<br><br>**Authors with competing interests**<br><br>Enter competing interest details beginning with this statement:<br><br>*I have read the journal's policy and the authors of this manuscript have the following competing interests: [insert competing interests here]*<br><br>* typeset	
**Ethics Statement**<br><br>Enter an ethics statement for this submission. This statement is required if the study involved:<br><br>• Human participants<br>• Human specimens or tissue<br>• Vertebrate animals or cephalopods<br>• Vertebrate embryos or tissues<br>• Field research<br><br>Write "N/A" if the submission does not require an ethics statement.<br><br>General guidance is provided below. Consult the submission guidelines for detailed instructions. **Make sure that all information entered here is included in the Methods section of the manuscript.**	Ethical approval for conducting this research was obtained from the Ministry of Health - Khartoum state / Directorate General Of Curative Medicine / No: 44, Sudan. Each respondent signs a consent before participation after providing a clear explanation. Data collection was anonymized, and the confidentiality of the study participants was maintained.

Powered by Editorial Manager® and ProduXion Manager® from Aries Systems Corporation

**Format for specific study types**<br><br>**Human Subject Research (involving human participants and/or tissue)**<br>• Give the name of the institutional review board or ethics committee that approved the study<br>• Include the approval number and/or a statement indicating approval of this research<br>• Indicate the form of consent obtained (written/oral) or the reason that consent was not obtained (e.g. the data were analyzed anonymously)<br><br>**Animal Research (involving vertebrate animals, embryos or tissues)**<br>• Provide the name of the Institutional Animal Care and Use Committee (IACUC) or other relevant ethics board that reviewed the study protocol, and indicate whether they approved this research or granted a formal waiver of ethical approval<br>• Include an approval number if one was obtained<br>• If the study involved *non-human primates*, add *additional details* about animal welfare and steps taken to ameliorate suffering<br>• If anesthesia, euthanasia, or any kind of animal sacrifice is part of the study, include briefly which substances and/or methods were applied<br><br>**Field Research**<br><br>Include the following details if this study involves the collection of plant, animal, or other materials from a natural setting:<br>• Field permit number<br>• Name of the institution or relevant body that granted permission	
**Data Availability**<br><br>Authors are required to make all data underlying the findings described fully available, without restriction, and from the time of publication. PLOS allows rare exceptions to address legal and ethical concerns. See the PLOS Data Policy and FAQ for detailed information.	Yes - all data are fully available without restriction

A Data Availability Statement describing where the data can be found is required at submission. Your answers to this question constitute the Data Availability Statement and **will be published in the article**, if accepted.<br><br>**Important:** Stating 'data available on request from the author' is not sufficient. If your data are only available upon request, select 'No' for the first question and explain your exceptional situation in the text box.<br><br>Do the authors confirm that all data underlying the findings described in their manuscript are fully available without restriction?	
**Describe where the data may be found in full sentences. If you are copying our sample text, replace any instances of XXX with the appropriate details.**<br><br>• If the data are **held or will be held in a public repository**, include URLs, accession numbers or DOIs. If this information will only be available after acceptance, indicate this by ticking the box below. For example: *All XXX files are available from the XXX database (accession number(s) XXX, XXX.)*.<br>• If the data are all contained **within the manuscript and/or Supporting Information files**, enter the following: *All relevant data are within the manuscript and its Supporting Information files.*<br>• If neither of these applies but you are able to provide **details of access elsewhere**, with or without limitations, please do so. For example:<br><br>*Data cannot be shared publicly because of [XXX]. Data are available from the XXX Institutional Data Access / Ethics Committee (contact via XXX) for researchers who meet the criteria for access to confidential data.*<br><br>*The data underlying the results presented in the study are available from (include the name of the third party*	The dataset generated during the current study is available from the corresponding author upon reasonable request.

and contact information or URL).<br>• This text is appropriate if the data are owned by a third party and authors do not have permission to share the data.<br><br>* typeset	
Additional data availability information:	

Manuscript

Click here to access/download;Manuscript;Revised Manuscript.docx

Burnout and its associated factors among healthcare workers in Khartoum's COVID-19 isolation centers: A cross-sectional study

Authors: Esraa S. A. Alfadul*[1], Malaz Mohammed Idrees Abdalmotalib[1], Salma Salah Khalid Alrawa[1], Rama Osman Abdelrahman Osman[1], Hadiea Mosaab AhmedElbashir Hassan[1], Alsamany taha albasheir[1], Elfatih A. Hasabo[1], Sagad O. O. Mohamed[1], Kamil Mirghani Ali Shaaban[2].

Affiliations

1. Faculty of Medicine, University of Khartoum, Khartoum, Sudan
2. Department of Community Medicine, Faculty of Medicine, University of Khartoum, Khartoum, Sudan

*__Corresponding Author__:

E-mail: Esraaalfadul.uofk@gmail.com (ESA)

ORCID: 0000-0001-9812-5095

Abstract

Background: Burnout prevalence and its consequences on healthcare workers during the Coronavirus Disease 2019 pandemic are not well investigated in Sudan. This study aims to assess the prevalence of burnout and its associated factors among doctors and nurses during the omicron wave in Khartoum isolation centers.

Method: A multi Centre facility-based cross-sectional study was conducted between 20th February and 10th April 2022 during the last wave of Coronavirus Disease 2019. A total of 306 doctors and nurses filled out the questionnaire, with a response rate of 64.8%. They were recruited from 5 isolation centers scattered in the three localities of Khartoum. The level of burnout was assessed using an online semi-structured questionnaire based on the Oldenburg Burnout Inventory questionnaire. Descriptive statistics were used for continuous variables and frequencies with percentages for categorical variables. The Chi-square test and Fisher exact test were used to identify variables associated with burnout. Logistic regression was used to determine the factors associated with burnout, and the p-value of ≤ .05 is considered statistically significant.

- **Results:** The prevalence of burnout was 45.7%. Multivariate logistic regression showed doctors were more likely to have burnout than nurses (OR:1,982, CI 95% 1,2173,228 p=0,006). Also, engaged healthcare workers were less likely to suffer from burnout than single healthcare workers (OR: 1.982, CI 95% 1.217 – 3.228; p =.006). A low number of household members (p= .017) was associated with burnout among participants.

Conclusion: There is a high prevalence of burnout among healthcare workers in Khartoum Isolation Centers, which is more apparent among doctors. Significant relations show a positive effect on burnout.

Introduction

The Coronavirus Disease 2019 (COVID-19) is a respiratory disease with varying degrees of morbidity and mortality depending on the age and fitness of the individual.[1] The virus emerged on 31st December 2019 in Wuhan, Hubei Province, China. [2] and has declared a global health emergency on 30th January 2020 and a global pandemic on 11th March 2020 by the World Health Organization.[3,4,5] Sudan reported the first COVID-19 case on 13th March 2020; since the declaration of the first case, the Sudan Federal Ministry of Health has enhanced the measures to combat the spread of the virus. On 13th April, after the registration of 10 cases of COVID-19, the authorities announced a total lockdown in the state of Khartoum - the most populated state in Sudan -suspending all gatherings, even prayers in mosques, as the virus has spread beyond Khartoum, reaching other states. On 25th May, five isolation centers were established in Khartoum to isolate and deal with COVID-19 cases. Later, the number of isolation centers increased in proportion to the high attack rate of the virus, reaching 14,401 cases with 1,116 deaths by 11th November 2020, reflecting a high fatality rate of 7.7%**.** By 7th July 2022, there were 62,745 confirmed cases of COVID-19, with 4,952 deaths in Sudan.[6- 9]

Although public health measures, such as lockdowns, and social distancing, are crucial to reducing the spread of COVID-19, they increase stress, anxiety, and mental disorders.[10] The World Health Organization has recognized "burnout" as an "occupational phenomenon ."As lockdowns have significantly affected our work-life balance and work environments, much research has ascertained burnout and its contributing factors.[11]

COVID-19 has a critical psychological impact on the community.[12] The length of the pandemic period- more than two years -, the shortage of data about the virus treatment, and the unknown destiny of the pandemic resulted in many studies that showed a significant increase in cases of anxiety, psychological stress, and depressive disorders worldwide, and because the medical staff worked under severe psychological pressure: being highly stressed by losing patients and colleagues, preference of having long shifts to protect their families, having no clear curing strategies, and urgency in instructing interpretation, all incredibly highlight peaking of burnout among healthcare providers.[13-16]

Burnout is a critical issue that generates inefficiency in healthcare organizations.[13] It lowers the quality of healthcare systems and negatively impacts patient prognosis.[17] Burnout affects the psychological well-being of the staff leading to medical errors. It makes the health system waste a lot of money and resources- because some workers leave their jobs, compelling the system to recruit new staff and offer a new training program. It impedes the process of psychological support for patients, which is part of the treatment.[17,18] Healthcare providers who are emotionally exhausted -express burnout- cannot support patients psychologically nor make fateful medical decisions.

While the pandemic affects the world, studies show that developed countries are affected less than developing ones. Africa is the most vulnerable region for the impact of higher mortality and morbidity, especially among healthcare providers.[16]

Sudan's revolution, the terrible economic status, collapsing health system, end-stage arrival of cases due to the centralized isolation centers, difficulty of transportation, and stigmatization of COVID-19 cases all affect the mental well-being of healthcare providers. Although, at present, no data considering the burnout among healthcare providers in Sudan, many published papers confirmed the high prevalence of burnout -among various countries during the pandemic due to diversified factors - leaving no doubt that the crisis is more exacerbated in Sudan.[19,20]

The term ‘Burnout’ entails emotional exhaustion, reduced feelings of achievement, and depersonalization [21]. According to studies, the prevalence of burnout among healthcare providers varies from 49.3% to 58% worldwide, and intensivists, emergency department doctors, and nurses are particularly susceptible [22]. These studies attributed the risk of burnout among healthcare providers to work-related, pandemic-related, and socio-demographic factors. A few examples include age, female gender, insufficient protective equipment, shortages in resources, stigma from the community, concerns about the financial situation, concerns about COVID-19, and workload and job demand [22].

Burnout prevalence and its consequences on healthcare workers are not well investigated in Sudan. Still, the stigmatization of mental problems in Sudan and the professional stigma of mental illness proposes that healthcare providers repress their mental suffering, navigating to more emotional stress and susceptibility to burnout.[23] Sudan’s health system has been facing critical challenges, including a lack of personal protective equipment and other necessary medical equipment. As a result, healthcare workers must deal with COVID-19 without the basic equipment to protect the patients and themselves from the infection. Consequently, this puts tremendous pressure on the doctors, largely due to the poor infection control policies and frustration over being unable to deliver the best possible patient care.[24]

The extent of burnout, its distribution between workers in different Isolation centers, and its predictors and protective factors are essential for decision-makers. In our study, we will measure the prevalence of burnout among healthcare providers exposed to COVID-19 patients, so we can contribute with data helping decision-makers fight the battle of the COVID-19 pandemic and the hidden pandemic -burnout -.

Material and Methods:

Study design and settings

This facility-based cross-sectional study was conducted between 20th February 2022 and 10th April 2020 during the last wave of COVID-19. There are ten isolation centers in Khartoum state distributed between the Sudanese Metropolis. These centers are Ibrahim Malik Teaching Hospital, Haj El-Mardi Hospital, Al-Shaab Teaching Hospital, Omdurman, Alnaw Hospital, Ahmed Gasim Teaching Hospital, Bahri Teaching Hospital, Jabra Hospital for emergency and injuries, Omdurman, and Khartoum Teaching Hospital.

We selected the largest and smallest isolation center from the three parts of the Sudanese metropolis. During the last wave, Ahmed Gasim Hospital was out of service, and there was one active isolation center in the city of Bahri. Therefore, the centers finally included in this study were Alnaw Hospital and Omdurman hospital from Omdurman, Al-Shaab Teaching Hospital, and Jabra Hospital for emergency and injuries from Khartoum, beside Bahri Teaching Hospital from Bahri.

Participants

The study participants were all doctors and nurses who worked in the selected centers during the study period. No one was excluded. We contacted Collaborators from each facility, and online surveys were supplied to them to disseminate to healthcare practitioners. The total number of the targeted healthcare practitioners was 472, and 306 completed the questionnaire, yielding a response rate of 64.8%

Data collection and tool

The burnout level of the participants was assessed using an online self-administered questionnaire. The questionnaire was divided into five sections, the first of which dealt with socio-demographic characteristics such as age, gender, residence, marital status, number of household members, presence of older family members in the household, history of comorbidities, and history of mental illness.

The second part was about the professional characteristics (isolation center, job title, years of experience, working in ICU, working hours and extra duties, and experience with COVID-19 infection); the third was related to predictors of burnout (COVID-19 among relatives, colleagues, and the patient himself, fear of infection, satisfaction with protective measures of the hospital, feeling of despair about COVID 19 patients). We also investigated adaptive behaviors among the participants (exercise, spirituality, smoking, professional support, self-care, mental breaks, and procrastination), and this was the fourth part of the questionnaire.

The fifth and final part assessed burnout and was based on the Oldenburg Burnout Inventory (OLBI) questionnaire. [25] It is a 16-item validated tool for the investigation of burnout. Items consist of positively and negatively worded questions related to exhaustion and disengagement that are recorded on a four-point Likert scale (from 1 = Strongly Agree to 4 = Strongly Disagree); one point designated the lowest burnout and four designated the highest. Disengagement refers to distancing oneself from the content of one's work, while exhaustion refers to fatigue, loss of energy, feelings of emptiness, and a strong need for rest. [26-28] Participants were considered at high risk of burnout if they met the thresholds of 2.1 or more for the exhaustion subscales and 2.25 or more for the disengagement subscales with a cut-off point of 35. [28]

Ethical approval

Ethical approval for conducting this research was obtained from the Ministry of Health - Khartoum state / Directorate General of Curative Medicine / No: 44, Sudan.

The participants were asked to give consent that they agree to participate in the study by filling the questionnaire for research purposes in the online form, and all the participants provided informed written

consent after providing a clear explanation of the study purpose. Data collection was anonymized, and the confidentiality of the study participants was maintained.

Statistical analysis plan

Data were extracted in an excel sheet, cleaned, and imported into the R software version 4.0.2 and SPSS version 28 (SPSS Inc., Chicago, IL, USA). The normality of distribution was tested using Kolmogorov-Smirnov test. Descriptive statistics were used in mean and Standard deviation for the continuous variables and frequencies with percentages for categorical variables. The Chi-square test and Fisher exact test were used to identify variables associated with burnout. Multiple logistic regression analyses evaluated the variables that showed a statistically significant relationship at the bivariate analysis level. The p-value of ≤ .05 was set as the significance level of the study.

Results

Participants information

A total of 306 healthcare workers, with an overall mean age of 26.7 ± 3.8 years participated in the study. More than half of the participants were males (n=156, 51.0%), and the majority were living in Omdurman (45.1%), followed by Khartoum (42.8%). Also, most of them were singles (83.3%), nurses (58.5%), and only 30 participants (9.8%) were suffering from comorbidities. Regarding the status of COVID-19 in their relatives, 38.6% stated that their relatives had been infected with COVID-19, while 17.0% were infected and then died**. (Table 1).**

Most respondents were from Jabra Isolation Center (38.6%) and Omdurman Teaching Hospital (33.3%). Among all participants, 22.5% had COVID-19, and nearly (53.6%) were working in the ICU. (**Table 1).**

Burnouts among participants

Responses to the Oldenburg Burnout Inventory (OLBI) are shown in **(Table 2).** Burnout prevalence was 45.7% among study participants. A low number of household members (p= 0.017) and being a doctor (p= 0.003) were associated with burnout among participants. Also, marital status was significantly associated with burnout (p = 0.001) **(Table 1).** Not surprisingly, fear of infection (p < 0.001) and satisfaction with hospital safety measures (p = 0.002) were associated with burnout.

The most frequent adaptive behaviors were taking brief mental breaks throughout the day (42.2%) and exercising (37.6%) (**Table 3).** Interestingly the following factors were not associated with burnout: average income (p >0.9), working years (p = 0.8), working hours (0.8), extra hours (p = 0.065), previous working experience in COVID-19 centers (p = 0.6) working center (p = 0.8) and site (p= 0.6) and fear of patient death despite all measure (p > 0.9)

The multivariate logistic regression showed doctors were more likely to have burnout than nurses (OR: 1.982, CI 95% 1.217 – 3.228; p = 0.006). Also, engaged healthcare workers were less likely to suffer burnout than unmarried healthcare workers (OR: 0.374, CI 95% 0.143– 0.980; P = 0.045) (**Table 4).**

Table 1: Baseline characteristics and demographic data of included participants.

Variables	N	Overall, N = 306[1]	Burnout		p-value[2]
			No, N = 166[1]	Yes, N = 140[1]	
**Age**	306	26.7 ± 3.8	26.8 ± 3.7	26.6 ± 4.0	0.7
**Gender**	306				0.8
Female		150 (49.0%)	80 (48.2%)	70 (50.0%)	
Male		156 (51.0%)	86 (51.8%)	70 (50.0%)	
**Residency**	306				0.9
Bahari		37 (12.1%)	19 (11.4%)	18 (12.9%)	
Khartoum		131 (42.8%)	70 (42.2%)	61 (43.6%)	
Omdurman		138 (45.1%)	77 (46.4%)	61 (43.6%)	
**Marital status**	306				**0.001**
Engaged		26 (8.5%)	20 (12.0%)	6 (4.3%)	
Married		22 (7.2%)	5 (3.0%)	17 (12.1%)	
Single		255 (83.3%)	140 (84.3%)	115 (82.1%)	
Widowed		3 (1.0%)	1 (0.6%)	2 (1.4%)	
**Number of household members**	299	6.4 ± 2.9	6.8 ± 2.5	6.0 ± 3.2	**0.017**
**Do you live with an elderly household member who has a chronic disease?**	306	131 (42.8%)	76 (45.8%)	55 (39.3%)	0.3
**Do you suffer from any comorbidities?**	306	30 (9.8%)	14 (8.4%)	16 (11.4%)	0.4
**Do you have a history of mental illness?**	306	7 (2.3%)	2 (1.2%)	5 (3.6%)	0.3
**Do you have relative/s who have been infected/died from Covid-19?**	306				0.7
Infected		118 (38.6%)	64 (38.6%)	54 (38.6%)	
Infected and died		52 (17.0%)	31 (18.7%)	21 (15.0%)	
None		136 (44.4%)	71 (42.8%)	65 (46.4%)	
**Do you have colleague/s who have been infected/died from Covid-19?**	306				0.8
Infected		230 (75.2%)	127 (76.5%)	103 (73.6%)	
Infected and died		17 (5.6%)	8 (4.8%)	9 (6.4%)	
None		59 (19.3%)	31 (18.7%)	28 (20.0%)	
**What was your COVID-19 Screening test result?**	306				0.088
Not Tested		131 (42.8%)	72 (43.4%)	59 (42.1%)	
Tested negative		106 (34.6%)	64 (38.6%)	42 (30.0%)	
Tested positive		69 (22.5%)	30 (18.1%)	39 (27.9%)	
**What is your Job title?**	306				**0.003**
Doctor		127 (41.5%)	56 (33.7%)	71 (50.7%)	
Nurse		179 (58.5%)	110 (66.3%)	69 (49.3%)	
**What is your perceived average income?**	306				>0.9
High		5 (1.6%)	3 (1.8%)	2 (1.4%)	
Low		116 (37.9%)	64 (38.6%)	52 (37.1%)	

Medium		185 (60.5%)	99 (59.6%)	86 (61.4%)	
**How many years have you been working in this job?**	306				0.3
(0-2)		218 (71.2%)	111 (66.9%)	107 (76.4%)	
(2-4)		51 (16.7%)	32 (19.3%)	19 (13.6%)	
(4-6)		24 (7.8%)	14 (8.4%)	10 (7.1%)	
(6-8)		4 (1.3%)	2 (1.2%)	2 (1.4%)	
(8-54)		9 (2.9%)	7 (4.2%)	2 (1.4%)	
**In which isolation center do you work?**	306				0.8
AlNaw Teaching Hospital		19 (6.2%)	12 (7.2%)	7 (5.0%)	
Al-Shaab Hospital		44 (14.4%)	21 (12.7%)	23 (16.4%)	
Bahri Hospital		23 (7.5%)	12 (7.2%)	11 (7.9%)	
Jebra Isolation center		118 (38.6%)	65 (39.2%)	53 (37.9%)	
Omdurman Teaching Hospital		102 (33.3%)	56 (33.7%)	46 (32.9%)	
**What was your way of employment?**	306				>0.9
Appointed by the Ministry of Health		49 (16.0%)	26 (15.7%)	23 (16.4%)	
Attachment		22 (7.2%)	13 (7.8%)	9 (6.4%)	
Compulsory service		19 (6.2%)	11 (6.6%)	8 (5.7%)	
Contracts: appointed by the hospital		216 (70.6%)	116 (69.9%)	100 (71.4%)	
**Did you work in isolation centers in the previous Covid-19 waves other than the current Omicron wave? Yes**	306	201 (65.7%)	111 (66.9%)	90 (64.3%)	0.6
**Do you work extra duty or extra hours per week? Yes**	306	194 (63.4%)	113 (68.1%)	81 (57.9%)	0.065
**In the isolation center, do you work in the ward or the intensive care unit (ICU)?**	306				0.6
ICU		164 (53.6%)	91 (54.8%)	73 (52.1%)	
The ward		142 (46.4%)	75 (45.2%)	67 (47.9%)	
**Working hours per week**	306	44.2 ± 18.9	44.5 ± 18.7	43.8 ± 19.2	0.8
**I'm afraid of getting infected by Covid-19 throughout my work.**	306				**<0.001**
1		85 (27.8%)	61 (36.7%)	24 (17.1%)	
2		44 (14.4%)	29 (17.5%)	15 (10.7%)	
3		99 (32.4%)	44 (26.5%)	55 (39.3%)	
4		43 (14.1%)	19 (11.4%)	24 (17.1%)	
5		35 (11.4%)	13 (7.8%)	22 (15.7%)	
**I feel safe with the protective measures taken by the hospital.**	306				**0.002**
1		71 (23.2%)	46 (27.7%)	25 (17.9%)	
2		77 (25.2%)	44 (26.5%)	33 (23.6%)	
3		90 (29.4%)	48 (28.9%)	42 (30.0%)	

4		41 (13.4%)	23 (13.9%)	18 (12.9%)	
5		27 (8.8%)	5 (3.0%)	22 (15.7%)	
**I feel the patient would die no matter what I did.**	306				>0.9
1		17 (5.6%)	10 (6.0%)	7 (5.0%)	
2		34 (11.1%)	19 (11.4%)	15 (10.7%)	
3		66 (21.6%)	38 (22.9%)	28 (20.0%)	
4		92 (30.1%)	49 (29.5%)	43 (30.7%)	
5		97 (31.7%)	50 (30.1%)	47 (33.6%)	

[1] Mean ± SD; n (%)

[2] Two Sample t-test; Pearson's Chi-squared test; Fisher's exact test

Note: 1 = Strongly Agree; 2 = Agree; 3 = Neutral; 4 = Disagree; 5 = Strongly Disagree

Table 2: Responses to questions for the burnout tool.

Variables	**Overall**, N = 306[1]	**Burnout**		**p-value**[2]
		**No**, N = 166[1]	**Yes**, N = 140[1]	
**I always find new and interesting aspects in my work.**				**<0.001**
1	113 (36.9%)	76 (45.8%)	37 (26.4%)	
2	118 (38.6%)	62 (37.3%)	56 (40.0%)	
3	51 (16.7%)	18 (10.8%)	33 (23.6%)	
4	24 (7.8%)	10 (6.0%)	14 (10.0%)	
**There are days when I feel tired before I arrive at work.**				**<0.001**
1	135 (44.1%)	94 (56.6%)	41 (29.3%)	
2	108 (35.3%)	56 (33.7%)	52 (37.1%)	
3	42 (13.7%)	9 (5.4%)	33 (23.6%)	
4	21 (6.9%)	7 (4.2%)	14 (10.0%)	
**It happens more and more often that I talk about my work in a negative way.**				**0.005**
1	62 (20.3%)	43 (25.9%)	19 (13.6%)	
2	99 (32.4%)	59 (35.5%)	40 (28.6%)	
3	77 (25.2%)	33 (19.9%)	44 (31.4%)	
4	68 (22.2%)	31 (18.7%)	37 (26.4%)	
**After work, I tend to need more time than in the past in order to relax and feel better.**				**<0.001**
1	137 (44.8%)	98 (59.0%)	39 (27.9%)	
2	92 (30.1%)	39 (23.5%)	53 (37.9%)	
3	52 (17.0%)	23 (13.9%)	29 (20.7%)	
4	25 (8.2%)	6 (3.6%)	19 (13.6%)	
**I can tolerate the pressure of my work very well.**				**<0.001**
1	113 (36.9%)	77 (46.4%)	36 (25.7%)	
2	121 (39.5%)	63 (38.0%)	58 (41.4%)	
3	54 (17.6%)	21 (12.7%)	33 (23.6%)	
4	18 (5.9%)	5 (3.0%)	13 (9.3%)	
**Lately, I tend to think less at work and do my job almost mechanically.**				**<0.001**
1	71 (23.2%)	57 (34.3%)	14 (10.0%)	
2	123 (40.2%)	68 (41.0%)	55 (39.3%)	
3	66 (21.6%)	22 (13.3%)	44 (31.4%)	
4	46 (15.0%)	19 (11.4%)	27 (19.3%)	
**I find my work to be a positive challenge.**				**<0.001**
1	131 (42.8%)	89 (53.6%)	42 (30.0%)	
2	109 (35.6%)	57 (34.3%)	52 (37.1%)	
3	48 (15.7%)	15 (9.0%)	33 (23.6%)	
4	18 (5.9%)	5 (3.0%)	13 (9.3%)	
**During my work, I often feel emotionally drained.**				**<0.001**
1	99 (32.4%)	72 (43.4%)	27 (19.3%)	
2	104 (34.0%)	59 (35.5%)	45 (32.1%)	
3	63 (20.6%)	18 (10.8%)	45 (32.1%)	
4	40 (13.1%)	17 (10.2%)	23 (16.4%)	
**Over time, one can become disconnected from this type of work.**				**<0.001**
1	93 (30.4%)	67 (40.4%)	26 (18.6%)	

2	110 (35.9%)	61 (36.7%)	49 (35.0%)	
3	70 (22.9%)	25 (15.1%)	45 (32.1%)	
4	33 (10.8%)	13 (7.8%)	20 (14.3%)	
**After working, I have enough energy for my leisure activities.**				0.3
1	38 (12.4%)	25 (15.1%)	13 (9.3%)	
2	79 (25.8%)	44 (26.5%)	35 (25.0%)	
3	77 (25.2%)	37 (22.3%)	40 (28.6%)	
4	112 (36.6%)	60 (36.1%)	52 (37.1%)	
**Sometimes I feel sickened by my work tasks.**				**<0.001**
1	83 (27.1%)	63 (38.0%)	20 (14.3%)	
2	109 (35.6%)	63 (38.0%)	46 (32.9%)	
3	81 (26.5%)	30 (18.1%)	51 (36.4%)	
4	33 (10.8%)	10 (6.0%)	23 (16.4%)	
**After my work, I usually feel worn out and weary.**				**<0.001**
1	133 (43.5%)	100 (60.2%)	33 (23.6%)	
2	97 (31.7%)	49 (29.5%)	48 (34.3%)	
3	56 (18.3%)	15 (9.0%)	41 (29.3%)	
4	20 (6.5%)	2 (1.2%)	18 (12.9%)	
**This is the only type of work that I can imagine myself doing.**				**<0.001**
1	57 (18.6%)	45 (27.1%)	12 (8.6%)	
2	67 (21.9%)	49 (29.5%)	18 (12.9%)	
3	106 (34.6%)	48 (28.9%)	58 (41.4%)	
4	76 (24.8%)	24 (14.5%)	52 (37.1%)	
**Usually, I can manage the amount of my work well.**				**<0.001**
1	112 (36.6%)	75 (45.2%)	37 (26.4%)	
2	140 (45.8%)	73 (44.0%)	67 (47.9%)	
3	41 (13.4%)	15 (9.0%)	26 (18.6%)	
4	13 (4.2%)	3 (1.8%)	10 (7.1%)	
**I feel more and more engaged in my work.**				**<0.001**
1	76 (24.8%)	63 (38.0%)	13 (9.3%)	
2	140 (45.8%)	79 (47.6%)	61 (43.6%)	
3	65 (21.2%)	18 (10.8%)	47 (33.6%)	
4	25 (8.2%)	6 (3.6%)	19 (13.6%)	
**When I work, I usually feel energized**				**<0.001**
1	79 (25.8%)	59 (35.5%)	20 (14.3%)	
2	131 (42.8%)	64 (38.6%)	67 (47.9%)	
3	67 (21.9%)	31 (18.7%)	36 (25.7%)	
4	29 (9.5%)	12 (7.2%)	17 (12.1%)	
**Score**	34.2 ± 5.3	30.4 ± 3.1	38.8 ± 3.5	**<0.001**

[1] n (%)

[2] Pearson's Chi-squared test; Fisher's exact test

Note: 1 = Strongly Agree; 2 = Agree; 3 = Disagree; 4 = Strongly Disagree

Table 3: Habits that participants have used.

Variables	**Overall**, N = 306[1]	**Burnout**		**p-value**[2]
		**No**, N = 166[1]	**Yes**, N = 140[1]	
**Exercise**	115 (37.6%)	61 (36.7%)	54 (38.6%)	0.7
**spiritual habits**	107 (35.0%)	63 (38.0%)	44 (31.4%)	0.2
**Smoking**	35 (11.4%)	18 (10.8%)	17 (12.1%)	0.7
**Seek professional support to cope with moral distress and grief**	41 (13.4%)	32 (19.3%)	9 (6.4%)	**0.001**
**Take brief mental breaks throughout the day**	129 (42.2%)	75 (45.2%)	54 (38.6%)	0.2
**Performing Self-care Routines**	108 (35.3%)	56 (33.7%)	52 (37.1%)	0.5
**Poor Time Management and Procrastination**	80 (26.1%)	49 (29.5%)	31 (22.1%)	0.14

[1] n (%)

[2] Pearson's Chi-squared test; Fisher's exact test

Table 4: Multivariate logistic regression for predictors of burnout among healthcare workers

**Variables**	**Multivariate Logistic regression**			
	**OR**	**95% CI**		**p-value**
		**Lower**	**Upper**	
**Marital status**				
Single	-	-	-	-
Married	3.974	1.367	11.547	0.011
Engaged	0.374	0.143	0.980	0.045
Widowed	2.289	0.199	26.317	0.506
**Number of household members**	0.948	0.867	1.036	0.241
**What is your Job title?**				
Nurse	-	-	-	-
Doctor	1.982	1.217	3.228	0.006

CI: Confidence Interval, OR= Odd ratio

Discussion

There is a considerable amount of evidence indicating the negative effects of COVID-19 on healthcare workers. Healthcare workers are experiencing increased psychological distress due to the COVID-19 pandemic, especially frontline staff. The current study addressed a significant issue and provided a better understanding of the problem in a low-income country. Approximately half of the healthcare workers in the COVID-19 isolation centers in Khartoum that were included in the present study suffered high levels of work-related stress and burnout in terms of emotional exhaustion and a lack of personal accomplishment and engagement. Physical and emotional well-being of health staff is critical to pandemic containment. However, high burnout rates pose significant threat to the delivery of safe and effective healthcare, and negatively influence healthcare providers, patients, and the healthcare system.

The prevalence of burnout reported in the current study is In line with previous studies assessing burnout among Sudanese healthcare workers during COVID-19. For instance, 71% of resident physicians in Sudan met the criteria for burnout using the OLBI tool [29]. Another study using the Maslach Burnout Inventory assessment tool revealed that 86.1% of resident physicians in Gezira State's teaching hospitals had burnout syndrome [30]. Further, these findings align with other countries that have reported high rates of burnout among healthcare workers during COVID-19 pandemic [31-35].

The high rates of burnout in this study can be explained by the psychological demands of the profession and the high level of socioeconomic pressure and work-related stress that lead healthcare workers to burnout [33]. The country's weak healthcare system, economic meltdown, security situation, political instability and conflicts during this period have placed more pressure on the healthcare system and professionals [34-35]. Moreover, dealing with a high number of patients with poor infrastructure , lack of suitable accommodation and transportation during the lockdown period, concerns about the lack of treatment supplies and personal protective equipment, and the fear of contracting the disease and infecting their families add to the problem.[36-37]. Nevertheless, COVID-19 has been ongoing for over two years, and the constant strain may exhaust healthcare workers' coping mechanisms.

Most of the socio-demographic and other variables assessed in this study were not statistically associated with burnout, reflecting the wide distribution of the problem among the participants despite such differences. We found that a lower number of household members, unmarried individuals, and physician jobs were the only variables associated with burnout among the participants. However, previous studies revealed inconsistent results on burnout risk factors among healthcare workers, and it is unclear whether that burnout follows a specific socio-demographic pattern among healthcare workers [29, 31, 35]. However, some of these associations with socio-demographic factors can be explainable. Among different medical professions, there are differences in the working duties, which put doctors at higher risk of violence and abuse. In our setting, doctors are the front-liners who deal with angry patients and their relatives, particularly when counseling them about their COVID-19 diagnosis and condition or when breaking the news that a patient has passed away [24, 37]. Furthermore, they are responsible for dealing with critically ill patients with a higher risk of complications and mortality [24].

Regarding the habits used to lessen burnout symptoms, seeking professional support to cope with moral distress and grief was significantly associated with lower rates of burnout among the participants. The participants have used other habits to alleviate the burnout symptoms, such as exercising, spiritual habits, and taking brief mental breaks throughout the day. However, none showed a significant association with burnout rates in this study.

The findings of this study acknowledge a high demand for appropriate support interventions and necessitate the search for more effective strategies to be implemented to reduce the risk of burnout among healthcare workers. Using different approaches to reduce burnout, such as training to improve stress-coping skills, can avoid or recover from emotional exhaustion and disengagement. In addition, managing the workplace and environment, improving systems, providing regular psychosocial support, and recruiting additional workers can help address organizational and workplace defects because hostile Working conditions contribute to stress and job dissatisfaction, resulting in depersonalization, emotional exhaustion, and lack of accomplishment [38,39].

Strengths and limitations

This is one of the few studies assessing the burnout burden among healthcare workers. The sample size of the participants, considered a low response population, is representative of generalizability to overall COVID-19 isolation centers in Khartoum, Sudan. The findings of this study need to be considered in the context of some limitations; as this is a cross-sectional study, it will be challenging to draw causative relationships. The self-reported nature of the study could raise the possibility of recall bias. Also, the study was done in five sites which might limit results generalization for all healthcare workers in all settings in the country and could compromise representativeness.

Conclusion

Our study has shown that nearly half of the healthcare workers in isolation centers in Khartoum have suffered from burnout during COVID-19. Several socio-demographic factors have contributed to the increased level of burnout, and multiple coping mechanisms have accounted for a lower level of burnout among healthcare workers. The findings of this study address the high demand for appropriate interventions to be implemented to reduce the risk of burnout among frontline healthcare workers.

Acknowledgment:

We want to thank Ahmed Abdalhalim Mohamed Omer, Omer Abdulmajed, Aya Zakareya Noor Hamid, Asmaa Mohamed Abbas, Aldoma Mohammed Adam Ismail, Elhadi elsiddig Elhadi, Mohammed AFEFE Hasan Salim, and Tagwa Badawe for their help in collecting the data and Mazin S. Haroun for his help in the research. Also, we would like to thank healthcare workers for participating in our study.

Contributors:

ESA, MMI, SSK, ROA, HMA, and ATA conceived and designed the study. EAH designed the statistical analysis plan, analyzed the data, and developed the figures and tables. ESA, MMI, SSK, ROA, HMA, ATA, EAH, and SOO reviewed and contributed to the interpretation. ESA, MMI, SSK, ROA, HMA, ATA, EAH, and SOO drafted the first version of the manuscript. KMA contributes to the study design and Supervision. All authors contributed to intellectual content during the drafting and revision of the work and approved the final version of the manuscript.

Funding:

The authors funded the research.

Data availability statement

The dataset generated during the current study is available upon request

Consent for publication:

All the authors gave their written and verbal consent for publication.

Conflict of interest:

The authors declare that they do not have any conflict of interest

References

1. Coronavirus disease (COVID-19) [Internet]. Who.int. [cited 2021 Dec 29]. Available from: https://www.who.int/health-topics/coronavirus
2. CDC. COVID-19 and your health [Internet]. Centers for Disease Control and Prevention. 2021 [cited 2021 Dec 29]. Available from: https://www.cdc.gov/coronavirus/2019-ncov/about/index.html
3. WHO declares public health emergency for novel Coronavirus [Internet]. Medscape. 2020 [cited 2021 Dec 29]. Available from: https://www.medscape.com/viewarticle/924596
4. Wee S-L, McNeil DG Jr, Hernández JC. W.H.o. Declares global emergency as Wuhan Coronavirus spreads. The New York Times [Internet]. 2020 30 January [cited 2021 29 December]. Available from: https://www.nytimes.com/2020/01/30/health/coronavirus-world-health-organization.html
5. Q5.U.S. to suspend most travel from Europe; N.b.a. pauses after player gets the virus. The New York Times [Internet]. 2020 Mar 11 [cited 2021 Dec 29]. Available from: https://www.nytimes.com/2020/03/11/world/coronavirus-news.html
6. SUDAN: Full lockdown in Khartoum. Africa Research Bulletin: Economic, Financial and Technical Series 2020 May;57. https://doi.org/10.1111/j.1467-6346.2020.09437.x
7. Kunna E. Sudan: Managing COVID-19 pandemic during a time of transition. Arab Reform Initiative [Internet]. 2020 [cited 2022 Jul 10]. Available from: https://www.arab-reform.net/publication/sudan-managing-covid-19-pandemic-during-a-time-of-transition/
8. Ahmed A, Mohamed NS, El-Sadig SM, Fahal LA, Abelrahim ZB, Ahmed ES, *et a*l. COVID-19 in Sudan. J Infect Dev Ctries. 2021 7 March;15:204-8. DOI: 10.3855/jidc.14520.
9. Sudan: WHO Coronavirus disease (COVID-19) dashboard with vaccination data [Internet]. Who.int. [cited 2022 Jul 10]. Available from: https://covid19.who.int/region/emro/country/sd
10. Coping with stress [Internet]. Cdc.gov. 2021 [cited 2021 Dec 29]. Available from: https://www.cdc.gov/mentalhealth/stress-coping/cope-with-stress/index.html
11. Burnout [Internet]. Mental Health UK. 2021 [cited 2021 Dec 29]. Available from: https://mentalhealth-uk.org/burnout/
12. Brooks SK, Webster RK, Smith LE, Woodland L, Wessely S, Greenberg N, *et al*. The psychological impact of quarantine and how to reduce it: a rapid review of the evidence. Lancet. 2020 Mar 14;395:912-20. DOI: 10.1016/S0140-6736(20)30460-8. Epub 2020 26 February.
13. Leo CG, Sabina S, Tumolo MR, Bodini A, Ponzini G, Sabato E, *et al*. Burnout Among Healthcare Workers in the COVID-19 Era: A Review of the Existing Literature. Front Public Health. 2021 29 October;9:750529. DOI: 10.3389/fpubh.2021.750529.
14. The Lancet. COVID-19: protecting healthcare workers. Lancet [Internet]. 2020 [cited 2021 Dec 29];395(10228):922. Available from: https://www.thelancet.com/journals/lancet/article/PIIS0140-6736(20)30644-9/fulltext
15. Søvold LE, Naslund JA, Kousoulis AA, Saxena S, Qoronfleh MW, Grobler C, *et al*. Prioritizing the mental health and well-being of healthcare workers: An urgent global public health priority. Front Public Health [Internet]. 2021 [cited 2021 Dec 29];9:679397. Available from: https://pubmed.ncbi.nlm.nih.gov/34026720/
16. Afulani PA, Gyamerah AO, Nutor JJ, Laar A, Aborigo RA, Malechi H, *et al*. Inadequate preparedness for response to COVID-19 is associated with stress and burnout among healthcare workers in Ghana. PLoS One. 2021 Apr 16;16:e0250294. DOI: 10.1371/journal.pone.0250294.

17. Patel RS, Bachu R, Adikey A, Malik M, Shah M. Factors related to physician burnout and its consequences: A review. Behav Sci (Basel) [Internet]. 2018;8:98. Available from: http://dx.doi.org/10.3390/bs8110098

18. Pélissier C, Charbotel B, Fassier JB, Fort E, Fontana L. Nurses' Occupational and Medical Risks Factors of Leaving the Profession in Nursing Homes. Int J Environ Res Public Health. 2018 Aug 27;15:1850. DOI: 10.3390/ijerph15091850.

19. Lin YY, Pan YA, Hsieh YL, Hsieh MH, Chuang YS, Hsu HY, *et al*. COVID-19 Pandemic Is Associated with an Adverse Impact on Burnout and Mood Disorder in Healthcare Professionals. Int J Environ Res Public Health. 2021 1 April;18:3654. DOI: 10.3390/ijerph18073654.

20. Nguyen J, Liu A, McKenney M, Liu H, Ang D, Elkbuli A. Impacts and challenges of the COVID-19 Pandemic on emergency medicine physicians in the United States. Am J Emerg Med. 2021 Oct;48:38-47. DOI: 10.1016/j.ajem.2021.03.088. Epub 2021 2 April.

21. Guastello AD, Brunson JC, Sambuco N, Dale LP, Tracy NA, Allen BR, Mathews CA. Predictors of professional burnout and fulfilment in a longitudinal analysis on nurses and healthcare workers in the COVID-19 pandemic. J Clin Nurs. 2022 Aug 10:10.1111/jocn.16463. doi: 10.1111/jocn.16463.

22. Gualano MR, Sinigaglia T, Lo Moro G, Rousset S, Cremona A, Bert F, Siliquini R. The Burden of Burnout among Healthcare Professionals of Intensive Care Units and Emergency Departments during the COVID-19 Pandemic: A Systematic Review. Int J Environ Res Public Health. 2021 Aug 2;18(15):8172. doi: 10.3390/ijerph18158172.

23. Brower KJ. Professional Stigma of Mental Health Issues: Physicians Are Both the Cause and Solution. Acad Med. 2021;96(5):635-40.

24. El-Sadig SM, Fahal LA, Abdelrahim ZB, Ahmed ES, Mohamed NS, Siddig EE. Impact of COVID-19 on doctors and healthcare providers during the pandemic in Sudan. Trans R Soc Trop Med Hyg. 2021;115(6):577-8.

25. Oldenburg Burnout Inventory (OLBI). (n.d.). MDApp. Retrieved 2 June, 2022, from https://www.mdapp.co/oldenburg-burnout-inventory-olbi-calculator-606/

26. Gruszczynska E, Basinska BA, Schaufeli WB. Within- and between-person factor structure of the Oldenburg Burnout Inventory: Analysis of a diary study using multilevel confirmatory factor analysis. PLOS ONE. 2021;16(5):e0251257.

27. Tipa RO, Tudose C, Pucarea VL. Measuring Burnout Among Psychiatric Residents Using the Oldenburg Burnout Inventory (OLBI) Instrument. J Med Life. 2019;12(4):354-60.

28. Summers RF, Gorrindo T, Hwang S, Aggarwal R, Guille C. Well-Being, Burnout, and Depression Among North American Psychiatrists: The State of Our Profession. Am J Psychiatry. 2020;177(10):955-64.

29. El Dabbah NA, Elhadi YAM. Burnout among frontline health professionals in two African countries: a cross-sectional study from Egypt and Sudan. 2021. doi: 10.21203/RS.3.RS-951355/V1

30. Elhadi YAM, Ahmed A, Salih EB, Abdelhamed OS, Ahmed MHH, El Dabbah NA. A cross-sectional survey of burnout in a sample of resident physicians in Sudan. PLOS ONE. 2022;17(3):e0265098.

31. Denning M, Goh ET, Tan B, Kanneganti A, Almonte M, Scott A, Martin G, Clarke J, Sounderajah V, et al. Determinants of burnout and other aspects of psychological well-being in healthcare workers during the Covid-19 Pandemic: A multinational cross-sectional study. PLoS One. 2021 Apr 16;16(4):e0238666. doi: 10.1371/journal.pone.0238666.

32. Elhadi M, Msherghi A, Elgzairi M, Alhashimi A, Bouhuwaish A, Biala M, Abuelmeda S, Khel S, Khaled A, Alsoufi A, Elmabrouk A, Alshiteewi FB, Hamed TB, Alhadi B, Alhaddad S, Elhadi A, Zaid A. Burnout Syndrome Among Hospital Healthcare Workers During the COVID-19 Pandemic and Civil War: A Cross-Sectional Study. Front Psychiatry. 2020 Dec 11;11:579563. doi: 10.3389/fpsyt.2020.579563.
33. Shoman Y, El May E, Marca S, Wild P, Bianchi R, Bugge M, et al. Predictors of occupational burnout: A systematic review. International Journal of Environmental Research and Public Health. 2021;18(17):9188
34. Sarboozi Hoseinabadi T, Kakhki S, Teimori G, Nayyeri S. Burnout and its influencing factors between frontline nurses and nurses from other wards during the outbreak of Coronavirus Disease -COVID-19- in Iran. Invest Educ Enferm. 2020 Jul;38(2):e3. DOI: 10.17533/udea.iee.v38n2e03.
35. Tan BYQ, Kanneganti A, Lim LJH, Tan M, Chua YX, Tan L, Sia CH, Denning M, Goh ET, Purkayastha S, Kinross J, Sim K, Chan YH, Ooi SBS. Burnout and Associated Factors Among Health Care Workers in Singapore During the COVID-19 Pandemic. J Am Med Dir Assoc. 2020 Dec;21(12):1751-1758.e5. doi: 10.1016/j.jamda.2020.09.035.
36. Elhadi M, Msherghi A, Elgzairi M, Alhashimi A, Bouhuwaish A, Biala M, Abuelmeda S, Khel S, Khaled A, Alsoufi A, Elhadi A, BenGhatnsh A. The Mental Well-Being of Frontline Physicians Working in Civil Wars Under Coronavirus Disease 2019 Pandemic Conditions. Front Psychiatry. 2021 Jan 14;11:598720. doi: 10.3389/fpsyt.2020.598720.
37. Elhadi YAM, Adebisi YA, Hassan KF, Mohammed SEE, Lin X, Lucero-Prisno Iii DE. The formidable task of fighting COVID-19 in Sudan. Pan Afr Med J. 2020 7 August;35(Suppl 2):137. doi: 10.11604/pamj.supp.2020.35.137.24984.
38. Pollock A, Campbell P, Cheyne J, Cowie J, Davis B, McCallum J, et al. Interventions to support the resilience and Mental Health of Frontline Health and social care professionals during and after a disease outbreak, epidemic or pandemic: A mixed methods systematic review. Cochrane Database of Systematic Reviews. 2020;2020(11).
39. Mengist B, Amha H, Ayenew T, Gedfew M, Akalu TY, Assemie MA, et al. Occupational stress and burnout among health care workers in Ethiopia: A systematic review and meta-analysis. Archives of Rehabilitation Research and Clinical Translation. 2021;3(2):100125.

The study's data.xlsx

Click here to access/download
Supporting Information
covid-19 burnout data.xlsx

Burnout and its associated factors among healthcare workers in Khartoum's COVID-19 isolation centers: A cross-sectional study

Authors: Esraa S. A. Alfadul*[1], Malaz Mohammed Idrees Abdalmotalib[1], Salma Salah Khalid Alrawa[1], Rama Osman Abdelrahman Osman[1], Hadiea Mosaab AhmedElbashir Hassan[1], Alsamany taha albasheir[1], Elfatih A. Hasabo[1], Sagad O. O. Mohamed[1], Kamil Mirghani Ali Shaaban[2].

Affiliations

1. Faculty of Medicine, University of Khartoum, Khartoum, Sudan
2. Department of Community Medicine, Faculty of Medicine, University of Khartoum, Khartoum, Sudan

*__Corresponding Author__:

E-mail: Esraaalfadul.uofk@gmail.com (ESA)

ORCID: 0000-0001-9812-5095

Abstract

Background: Burnout prevalence and its consequences on healthcare workers during the Coronavirus Disease 2019 pandemic are not well investigated in Sudan. This study aims to assess the prevalence of burnout and its associated factors among doctors and nurses during the omicron wave in Khartoum isolation centers.

Method: A multi Centre facility-based cross-sectional study was conducted between 20th February and 10th April 2022 during the last wave of Coronavirus Disease 2019. A total of 306 doctors and nurses filled out the questionnaire, with a response rate of 64.8%. They were recruited from 5 isolation centers scattered in the three localities of Khartoum. The level of burnout was assessed using an online semi-structured questionnaire based on the Oldenburg Burnout Inventory questionnaire. Descriptive statistics were used for continuous variables and frequencies with percentages for categorical variables. The Chi-square test and Fisher exact test were used to identify variables associated with burnout. Logistic regression was used to determine the factors associated with burnout, and the p-value of ≤ .05 is considered statistically significant.

- **Results:** The prevalence of burnout was 45.7%. Multivariate logistic regression showed doctors were more likely to have burnout than nurses (OR:1,982, CI 95% 1,2173,228 p=0,006). Also, engaged healthcare workers were less likely to suffer from burnout than single healthcare workers (OR: 1.982, CI 95% 1.217 – 3.228; p =.006). A low number of household members (p= .017) was associated with burnout among participants.

Conclusion: There is a high prevalence of burnout among healthcare workers in Khartoum Isolation Centers, which is more apparent among doctors. Significant relations show a positive effect on burnout.

Introduction

The Coronavirus Disease 2019 (COVID-19) is a respiratory disease with varying degrees of morbidity and mortality depending on the age and fitness of the individual.[1] The virus emerged on 31st December 2019 in Wuhan, Hubei Province, China. [2] and has declared a global health emergency on 30th January 2020 and a global pandemic on 11th March 2020 by the World Health Organization.[3,4,5] Sudan reported the first COVID-19 case on 13th March 2020; since the declaration of the first case, the Sudan Federal Ministry of Health has enhanced the measures to combat the spread of the virus. On 13th April, after the registration of 10 cases of COVID-19, the authorities announced a total lockdown in the state of Khartoum - the most populated state in Sudan -suspending all gatherings, even prayers in mosques, as the virus has spread beyond Khartoum, reaching other states. On 25th May, five isolation centers were established in Khartoum to isolate and deal with COVID-19 cases. Later, the number of isolation centers increased in proportion to the high attack rate of the virus, reaching 14,401 cases with 1,116 deaths by 11th November 2020, reflecting a high fatality rate of 7.7%**.** By 7th July 2022, there were 62,745 confirmed cases of COVID-19, with 4,952 deaths in Sudan.[6- 9]

Although public health measures, such as lockdowns, and social distancing, are crucial to reducing the spread of COVID-19, they increase stress, anxiety, and mental disorders.[10] The World Health Organization has recognized "burnout" as an "occupational phenomenon ."As lockdowns have significantly affected our work-life balance and work environments, much research has ascertained burnout and its contributing factors.[11]

COVID-19 has a critical psychological impact on the community.[12] The length of the pandemic period-more than two years -, the shortage of data about the virus treatment, and the unknown destiny of the pandemic resulted in many studies that showed a significant increase in cases of anxiety, psychological stress, and depressive disorders worldwide, and because the medical staff worked under severe psychological pressure: being highly stressed by losing patients and colleagues, preference of having long shifts to protect their families, having no clear curing strategies, and urgency in instructing interpretation, all incredibly highlight peaking of burnout among healthcare providers.[13-16]

Burnout is a critical issue that generates inefficiency in healthcare organizations.[13] It lowers the quality of healthcare systems and negatively impacts patient prognosis.[17] Burnout affects the psychological well-being of the staff leading to medical errors. It makes the health system waste a lot of money and resources-because some workers leave their jobs, compelling the system to recruit new staff and offer a new training program. It impedes the process of psychological support for patients, which is part of the treatment.[17,18] Healthcare providers who are emotionally exhausted -express burnout- cannot support patients psychologically nor make fateful medical decisions.

While the pandemic affects the world, studies show that developed countries are affected less than developing ones. Africa is the most vulnerable region for the impact of higher mortality and morbidity, especially among healthcare providers.[16]

Sudan's revolution, the terrible economic status, collapsing health system, end-stage arrival of cases due to the centralized isolation centers, difficulty of transportation, and stigmatization of COVID-19 cases all affect the mental well-being of healthcare providers. Although, at present, no data considering the burnout among healthcare providers in Sudan, many published papers confirmed the high prevalence of burnout -among various countries during the pandemic due to diversified factors - leaving no doubt that the crisis is more exacerbated in Sudan.[19,20]

The term ‘Burnout’ entails emotional exhaustion, reduced feelings of achievement, and depersonalization [21]. According to studies, the prevalence of burnout among healthcare providers varies from 49.3% to 58% worldwide, and intensivists, emergency department doctors, and nurses are particularly susceptible [22]. These studies attributed the risk of burnout among healthcare providers to work-related, pandemic-related, and socio-demographic factors. A few examples include age, female gender, insufficient protective equipment, shortages in resources, stigma from the community, concerns about the financial situation, concerns about COVID-19, and workload and job demand [22].

Burnout prevalence and its consequences on healthcare workers are not well investigated in Sudan. Still, the stigmatization of mental problems in Sudan and the professional stigma of mental illness proposes that healthcare providers repress their mental suffering, navigating to more emotional stress and susceptibility to burnout.[23] Sudan’s health system has been facing critical challenges, including a lack of personal protective equipment and other necessary medical equipment. As a result, healthcare workers must deal with COVID-19 without the basic equipment to protect the patients and themselves from the infection. Consequently, this puts tremendous pressure on the doctors, largely due to the poor infection control policies and frustration over being unable to deliver the best possible patient care.[24]

The extent of burnout, its distribution between workers in different Isolation centers, and its predictors and protective factors are essential for decision-makers. In our study, we will measure the prevalence of burnout among healthcare providers exposed to COVID-19 patients, so we can contribute with data helping decision-makers fight the battle of the COVID-19 pandemic and the hidden pandemic -burnout -.

Material and Methods:

Study design and settings

This facility-based cross-sectional study was conducted between 20th February 2022 and 10th April 2020 during the last wave of COVID-19. There are ten isolation centers in Khartoum state distributed between the Sudanese Metropolis. These centers are Ibrahim Malik Teaching Hospital, Haj El-Mardi Hospital, Al-Shaab Teaching Hospital, Omdurman, Alnaw Hospital, Ahmed Gasim Teaching Hospital, Bahri Teaching Hospital, Jabra Hospital for emergency and injuries, Omdurman, and Khartoum Teaching Hospital.

We selected the largest and smallest isolation center from the three parts of the Sudanese metropolis. During the last wave, Ahmed Gasim Hospital was out of service, and there was one active isolation center in the city of Bahri. Therefore, the centers finally included in this study were Alnaw Hospital and Omdurman hospital from Omdurman, Al-Shaab Teaching Hospital, and Jabra Hospital for emergency and injuries from Khartoum, beside Bahri Teaching Hospital from Bahri.

Participants

The study participants were all doctors and nurses who worked in the selected centers during the study period. No one was excluded. We contacted Collaborators from each facility, and online surveys were supplied to them to disseminate to healthcare practitioners. The total number of the targeted healthcare practitioners was 472, and 306 completed the questionnaire, yielding a response rate of 64.8%

Data collection and tool

The burnout level of the participants was assessed using an online self-administered questionnaire. The questionnaire was divided into five sections, the first of which dealt with socio-demographic characteristics such as age, gender, residence, marital status, number of household members, presence of older family members in the household, history of comorbidities, and history of mental illness.

The second part was about the professional characteristics (isolation center, job title, years of experience, working in ICU, working hours and extra duties, and experience with COVID-19 infection); the third was related to predictors of burnout (COVID-19 among relatives, colleagues, and the patient himself, fear of infection, satisfaction with protective measures of the hospital, feeling of despair about COVID 19 patients). We also investigated adaptive behaviors among the participants (exercise, spirituality, smoking, professional support, self-care, mental breaks, and procrastination), and this was the fourth part of the questionnaire.

The fifth and final part assessed burnout and was based on the Oldenburg Burnout Inventory (OLBI) questionnaire. [25] It is a 16-item validated tool for the investigation of burnout. Items consist of positively and negatively worded questions related to exhaustion and disengagement that are recorded on a four-point Likert scale (from 1 = Strongly Agree to 4 = Strongly Disagree); one point designated the lowest burnout and four designated the highest. Disengagement refers to distancing oneself from the content of one's work, while exhaustion refers to fatigue, loss of energy, feelings of emptiness, and a strong need for rest. [26-28] Participants were considered at high risk of burnout if they met the thresholds of 2.1 or more for the exhaustion subscales and 2.25 or more for the disengagement subscales with a cut-off point of 35. [28]

Ethical approval

Ethical approval for conducting this research was obtained from the Ministry of Health - Khartoum state / Directorate General of Curative Medicine / No: 44, Sudan.

The participants were asked to give consent that they agree to participate in the study by filling the questionnaire for research purposes in the online form, and all the participants provided informed written

consent after providing a clear explanation of the study purpose. Data collection was anonymized, and the confidentiality of the study participants was maintained.

Statistical analysis plan

Data were extracted in an excel sheet, cleaned, and imported into the R software version 4.0.2 and SPSS version 28 (SPSS Inc., Chicago, IL, USA). The normality of distribution was tested using Kolmogorov-Smirnov test. Descriptive statistics were used in mean and Standard deviation for the continuous variables and frequencies with percentages for categorical variables. The Chi-square test and Fisher exact test were used to identify variables associated with burnout. Multiple logistic regression analyses evaluated the variables that showed a statistically significant relationship at the bivariate analysis level. The p-value of ≤ .05 was set as the significance level of the study.

Results
Participants information

A total of 306 healthcare workers, with an overall mean age of 26.7 ± 3.8 years participated in the study. More than half of the participants were males (n=156, 51.0%), and the majority were living in Omdurman (45.1%), followed by Khartoum (42.8%). Also, most of them were singles (83.3%), nurses (58.5%), and only 30 participants (9.8%) were suffering from comorbidities. Regarding the status of COVID-19 in their relatives, 38.6% stated that their relatives had been infected with COVID-19, while 17.0% were infected and then died**. (Table 1).**

Most respondents were from Jabra Isolation Center (38.6%) and Omdurman Teaching Hospital (33.3%). Among all participants, 22.5% had COVID-19, and nearly (53.6%) were working in the ICU. (**Table 1).**

Burnouts among participants

Responses to the Oldenburg Burnout Inventory (OLBI) are shown in **(Table 2).** Burnout prevalence was 45.7% among study participants. A low number of household members (p= 0.017) and being a doctor (p= 0.003) were associated with burnout among participants. Also, marital status was significantly associated with burnout (p = 0.001) **(Table 1).** Not surprisingly, fear of infection (p < 0.001) and satisfaction with hospital safety measures (p = 0.002) were associated with burnout.

The most frequent adaptive behaviors were taking brief mental breaks throughout the day (42.2%) and exercising (37.6%) (**Table 3).** Interestingly the following factors were not associated with burnout: average income (p >0.9), working years (p = 0.8), working hours (0.8), extra hours (p = 0.065), previous working experience in COVID-19 centers (p = 0.6) working center (p = 0.8) and site (p= 0.6) and fear of patient death despite all measure (p > 0.9)

The multivariate logistic regression showed doctors were more likely to have burnout than nurses (OR: 1.982, CI 95% 1.217 – 3.228; p = 0.006). Also, engaged healthcare workers were less likely to suffer burnout than unmarried healthcare workers (OR: 0.374, CI 95% 0.143– 0.980; P = 0.045) (**Table 4).**

Table 1: Baseline characteristics and demographic data of included participants.

Variables	N	**Overall**, N = 306[1]	**Burnout** **No**, N = 166[1]	**Burnout** **Yes**, N = 140[1]	**p-value**[2]
**Age**	306	26.7 ± 3.8	26.8 ± 3.7	26.6 ± 4.0	0.7
**Gender**	306				0.8
Female		150 (49.0%)	80 (48.2%)	70 (50.0%)	
Male		156 (51.0%)	86 (51.8%)	70 (50.0%)	
**Residency**	306				0.9
Bahari		37 (12.1%)	19 (11.4%)	18 (12.9%)	
Khartoum		131 (42.8%)	70 (42.2%)	61 (43.6%)	
Omdurman		138 (45.1%)	77 (46.4%)	61 (43.6%)	
**Marital status**	306				**0.001**
Engaged		26 (8.5%)	20 (12.0%)	6 (4.3%)	
Married		22 (7.2%)	5 (3.0%)	17 (12.1%)	
Single		255 (83.3%)	140 (84.3%)	115 (82.1%)	
Widowed		3 (1.0%)	1 (0.6%)	2 (1.4%)	
**Number of household members**	299	6.4 ± 2.9	6.8 ± 2.5	6.0 ± 3.2	**0.017**
**Do you live with an elderly household member who has a chronic disease?**	306	131 (42.8%)	76 (45.8%)	55 (39.3%)	0.3
**Do you suffer from any comorbidities?**	306	30 (9.8%)	14 (8.4%)	16 (11.4%)	0.4
**Do you have a history of mental illness?**	306	7 (2.3%)	2 (1.2%)	5 (3.6%)	0.3
**Do you have relative/s who have been infected/died from Covid-19?**	306				0.7
Infected		118 (38.6%)	64 (38.6%)	54 (38.6%)	
Infected and died		52 (17.0%)	31 (18.7%)	21 (15.0%)	
None		136 (44.4%)	71 (42.8%)	65 (46.4%)	
**Do you have colleague/s who have been infected/died from Covid-19?**	306				0.8
Infected		230 (75.2%)	127 (76.5%)	103 (73.6%)	
Infected and died		17 (5.6%)	8 (4.8%)	9 (6.4%)	
None		59 (19.3%)	31 (18.7%)	28 (20.0%)	
**What was your COVID-19 Screening test result?**	306				0.088
Not Tested		131 (42.8%)	72 (43.4%)	59 (42.1%)	
Tested negative		106 (34.6%)	64 (38.6%)	42 (30.0%)	
Tested positive		69 (22.5%)	30 (18.1%)	39 (27.9%)	
**What is your Job title?**	306				**0.003**
Doctor		127 (41.5%)	56 (33.7%)	71 (50.7%)	
Nurse		179 (58.5%)	110 (66.3%)	69 (49.3%)	
**What is your perceived average income?**	306				>0.9
High		5 (1.6%)	3 (1.8%)	2 (1.4%)	
Low		116 (37.9%)	64 (38.6%)	52 (37.1%)	

Medium		185 (60.5%)	99 (59.6%)	86 (61.4%)	
**How many years have you been working in this job?**	306				0.3
(0-2)		218 (71.2%)	111 (66.9%)	107 (76.4%)	
(2-4)		51 (16.7%)	32 (19.3%)	19 (13.6%)	
(4-6)		24 (7.8%)	14 (8.4%)	10 (7.1%)	
(6-8)		4 (1.3%)	2 (1.2%)	2 (1.4%)	
(8-54)		9 (2.9%)	7 (4.2%)	2 (1.4%)	
**In which isolation center do you work?**	306				0.8
AlNaw Teaching Hospital		19 (6.2%)	12 (7.2%)	7 (5.0%)	
Al-Shaab Hospital		44 (14.4%)	21 (12.7%)	23 (16.4%)	
Bahri Hospital		23 (7.5%)	12 (7.2%)	11 (7.9%)	
Jebra Isolation center		118 (38.6%)	65 (39.2%)	53 (37.9%)	
Omdurman Teaching Hospital		102 (33.3%)	56 (33.7%)	46 (32.9%)	
**What was your way of employment?**	306				>0.9
Appointed by the Ministry of Health		49 (16.0%)	26 (15.7%)	23 (16.4%)	
Attachment		22 (7.2%)	13 (7.8%)	9 (6.4%)	
Compulsory service		19 (6.2%)	11 (6.6%)	8 (5.7%)	
Contracts: appointed by the hospital		216 (70.6%)	116 (69.9%)	100 (71.4%)	
**Did you work in isolation centers in the previous Covid-19 waves other than the current Omicron wave? Yes**	306	201 (65.7%)	111 (66.9%)	90 (64.3%)	0.6
**Do you work extra duty or extra hours per week? Yes**	306	194 (63.4%)	113 (68.1%)	81 (57.9%)	0.065
**In the isolation center, do you work in the ward or the intensive care unit (ICU)?**	306				0.6
ICU		164 (53.6%)	91 (54.8%)	73 (52.1%)	
The ward		142 (46.4%)	75 (45.2%)	67 (47.9%)	
**Working hours per week**	306	44.2 ± 18.9	44.5 ± 18.7	43.8 ± 19.2	0.8
**I'm afraid of getting infected by Covid-19 throughout my work.**	306				**<0.001**
1		85 (27.8%)	61 (36.7%)	24 (17.1%)	
2		44 (14.4%)	29 (17.5%)	15 (10.7%)	
3		99 (32.4%)	44 (26.5%)	55 (39.3%)	
4		43 (14.1%)	19 (11.4%)	24 (17.1%)	
5		35 (11.4%)	13 (7.8%)	22 (15.7%)	
**I feel safe with the protective measures taken by the hospital.**	306				**0.002**
1		71 (23.2%)	46 (27.7%)	25 (17.9%)	
2		77 (25.2%)	44 (26.5%)	33 (23.6%)	
3		90 (29.4%)	48 (28.9%)	42 (30.0%)	

4		41 (13.4%)	23 (13.9%)	18 (12.9%)	
5		27 (8.8%)	5 (3.0%)	22 (15.7%)	
**I feel the patient would die no matter what I did.**	306				>0.9
1		17 (5.6%)	10 (6.0%)	7 (5.0%)	
2		34 (11.1%)	19 (11.4%)	15 (10.7%)	
3		66 (21.6%)	38 (22.9%)	28 (20.0%)	
4		92 (30.1%)	49 (29.5%)	43 (30.7%)	
5		97 (31.7%)	50 (30.1%)	47 (33.6%)	

[1] Mean ± SD; n (%)

[2] Two Sample t-test; Pearson's Chi-squared test; Fisher's exact test

Note: 1 = Strongly Agree; 2 = Agree; 3 = Neutral; 4 = Disagree; 5 = Strongly Disagree

Table 2: Responses to questions for the burnout tool.

Variables	**Overall**, N = 306[1]	**Burnout** **No**, N = 166[1]	**Yes**, N = 140[1]	**p-value**[2]
**I always find new and interesting aspects in my work.**				**<0.001**
1	113 (36.9%)	76 (45.8%)	37 (26.4%)	
2	118 (38.6%)	62 (37.3%)	56 (40.0%)	
3	51 (16.7%)	18 (10.8%)	33 (23.6%)	
4	24 (7.8%)	10 (6.0%)	14 (10.0%)	
**There are days when I feel tired before I arrive at work.**				**<0.001**
1	135 (44.1%)	94 (56.6%)	41 (29.3%)	
2	108 (35.3%)	56 (33.7%)	52 (37.1%)	
3	42 (13.7%)	9 (5.4%)	33 (23.6%)	
4	21 (6.9%)	7 (4.2%)	14 (10.0%)	
**It happens more and more often that I talk about my work in a negative way.**				**0.005**
1	62 (20.3%)	43 (25.9%)	19 (13.6%)	
2	99 (32.4%)	59 (35.5%)	40 (28.6%)	
3	77 (25.2%)	33 (19.9%)	44 (31.4%)	
4	68 (22.2%)	31 (18.7%)	37 (26.4%)	
**After work, I tend to need more time than in the past in order to relax and feel better.**				**<0.001**
1	137 (44.8%)	98 (59.0%)	39 (27.9%)	
2	92 (30.1%)	39 (23.5%)	53 (37.9%)	
3	52 (17.0%)	23 (13.9%)	29 (20.7%)	
4	25 (8.2%)	6 (3.6%)	19 (13.6%)	
**I can tolerate the pressure of my work very well.**				**<0.001**
1	113 (36.9%)	77 (46.4%)	36 (25.7%)	
2	121 (39.5%)	63 (38.0%)	58 (41.4%)	
3	54 (17.6%)	21 (12.7%)	33 (23.6%)	
4	18 (5.9%)	5 (3.0%)	13 (9.3%)	
**Lately, I tend to think less at work and do my job almost mechanically.**				**<0.001**
1	71 (23.2%)	57 (34.3%)	14 (10.0%)	
2	123 (40.2%)	68 (41.0%)	55 (39.3%)	
3	66 (21.6%)	22 (13.3%)	44 (31.4%)	
4	46 (15.0%)	19 (11.4%)	27 (19.3%)	
**I find my work to be a positive challenge.**				**<0.001**
1	131 (42.8%)	89 (53.6%)	42 (30.0%)	
2	109 (35.6%)	57 (34.3%)	52 (37.1%)	
3	48 (15.7%)	15 (9.0%)	33 (23.6%)	
4	18 (5.9%)	5 (3.0%)	13 (9.3%)	
**During my work, I often feel emotionally drained.**				**<0.001**
1	99 (32.4%)	72 (43.4%)	27 (19.3%)	
2	104 (34.0%)	59 (35.5%)	45 (32.1%)	
3	63 (20.6%)	18 (10.8%)	45 (32.1%)	
4	40 (13.1%)	17 (10.2%)	23 (16.4%)	
**Over time, one can become disconnected from this type of work.**				**<0.001**
1	93 (30.4%)	67 (40.4%)	26 (18.6%)	

2	110 (35.9%)	61 (36.7%)	49 (35.0%)	
3	70 (22.9%)	25 (15.1%)	45 (32.1%)	
4	33 (10.8%)	13 (7.8%)	20 (14.3%)	
**After working, I have enough energy for my leisure activities.**				0.3
1	38 (12.4%)	25 (15.1%)	13 (9.3%)	
2	79 (25.8%)	44 (26.5%)	35 (25.0%)	
3	77 (25.2%)	37 (22.3%)	40 (28.6%)	
4	112 (36.6%)	60 (36.1%)	52 (37.1%)	
**Sometimes I feel sickened by my work tasks.**				**<0.001**
1	83 (27.1%)	63 (38.0%)	20 (14.3%)	
2	109 (35.6%)	63 (38.0%)	46 (32.9%)	
3	81 (26.5%)	30 (18.1%)	51 (36.4%)	
4	33 (10.8%)	10 (6.0%)	23 (16.4%)	
**After my work, I usually feel worn out and weary.**				**<0.001**
1	133 (43.5%)	100 (60.2%)	33 (23.6%)	
2	97 (31.7%)	49 (29.5%)	48 (34.3%)	
3	56 (18.3%)	15 (9.0%)	41 (29.3%)	
4	20 (6.5%)	2 (1.2%)	18 (12.9%)	
**This is the only type of work that I can imagine myself doing.**				**<0.001**
1	57 (18.6%)	45 (27.1%)	12 (8.6%)	
2	67 (21.9%)	49 (29.5%)	18 (12.9%)	
3	106 (34.6%)	48 (28.9%)	58 (41.4%)	
4	76 (24.8%)	24 (14.5%)	52 (37.1%)	
**Usually, I can manage the amount of my work well.**				**<0.001**
1	112 (36.6%)	75 (45.2%)	37 (26.4%)	
2	140 (45.8%)	73 (44.0%)	67 (47.9%)	
3	41 (13.4%)	15 (9.0%)	26 (18.6%)	
4	13 (4.2%)	3 (1.8%)	10 (7.1%)	
**I feel more and more engaged in my work.**				**<0.001**
1	76 (24.8%)	63 (38.0%)	13 (9.3%)	
2	140 (45.8%)	79 (47.6%)	61 (43.6%)	
3	65 (21.2%)	18 (10.8%)	47 (33.6%)	
4	25 (8.2%)	6 (3.6%)	19 (13.6%)	
**When I work, I usually feel energized**				**<0.001**
1	79 (25.8%)	59 (35.5%)	20 (14.3%)	
2	131 (42.8%)	64 (38.6%)	67 (47.9%)	
3	67 (21.9%)	31 (18.7%)	36 (25.7%)	
4	29 (9.5%)	12 (7.2%)	17 (12.1%)	
**Score**	34.2 ± 5.3	30.4 ± 3.1	38.8 ± 3.5	**<0.001**

[1] n (%)

[2] Pearson's Chi-squared test; Fisher's exact test

Note: 1 = Strongly Agree; 2 = Agree; 3 = Disagree; 4 = Strongly Disagree

Table 3: Habits that participants have used.

Variables	**Overall**, N = 306[1]	**Burnout** **No**, N = 166[1]	**Yes**, N = 140[1]	**p-value**[2]
**Exercise**	115 (37.6%)	61 (36.7%)	54 (38.6%)	0.7
**spiritual habits**	107 (35.0%)	63 (38.0%)	44 (31.4%)	0.2
**Smoking**	35 (11.4%)	18 (10.8%)	17 (12.1%)	0.7
**Seek professional support to cope with moral distress and grief**	41 (13.4%)	32 (19.3%)	9 (6.4%)	**0.001**
**Take brief mental breaks throughout the day**	129 (42.2%)	75 (45.2%)	54 (38.6%)	0.2
**Performing Self-care Routines**	108 (35.3%)	56 (33.7%)	52 (37.1%)	0.5
**Poor Time Management and Procrastination**	80 (26.1%)	49 (29.5%)	31 (22.1%)	0.14

[1] n (%)

[2] Pearson's Chi-squared test; Fisher's exact test

Table 4: Multivariate logistic regression for predictors of burnout among healthcare workers

Variables	Multivariate Logistic regression			
	OR	95% CI		p-value
		Lower	Upper	
**Marital status**				
Single	-	-	-	-
Married	3.974	1.367	11.547	0.011
Engaged	0.374	0.143	0.980	0.045
Widowed	2.289	0.199	26.317	0.506
**Number of household members**	0.948	0.867	1.036	0.241
**What is your Job title?**				
Nurse	-	-	-	-
Doctor	1.982	1.217	3.228	0.006

CI: Confidence Interval, OR= Odd ratio

Discussion

There is a considerable amount of evidence indicating the negative effects of COVID-19 on healthcare workers. Healthcare workers are experiencing increased psychological distress due to the COVID-19 pandemic, especially frontline staff. The current study addressed a significant issue and provided a better understanding of the problem in a low-income country. Approximately half of the healthcare workers in the COVID-19 isolation centers in Khartoum that were included in the present study suffered high levels of work-related stress and burnout in terms of emotional exhaustion and a lack of personal accomplishment and engagement. Physical and emotional well-being of health staff is critical to pandemic containment. However, high burnout rates pose significant threat to the delivery of safe and effective healthcare, and negatively influence healthcare providers, patients, and the healthcare system.

The prevalence of burnout reported in the current study is In line with previous studies assessing burnout among Sudanese healthcare workers during COVID-19. For instance, 71% of resident physicians in Sudan met the criteria for burnout using the OLBI tool [29]. Another study using the Maslach Burnout Inventory assessment tool revealed that 86.1% of resident physicians in Gezira State's teaching hospitals had burnout syndrome [30]. Further, these findings align with other countries that have reported high rates of burnout among healthcare workers during COVID-19 pandemic [31-35].

The high rates of burnout in this study can be explained by the psychological demands of the profession and the high level of socioeconomic pressure and work-related stress that lead healthcare workers to burnout [33]. The country's weak healthcare system, economic meltdown, security situation, political instability and conflicts during this period have placed more pressure on the healthcare system and professionals [34-35]. Moreover, dealing with a high number of patients with poor infrastructure , lack of suitable accommodation and transportation during the lockdown period, concerns about the lack of treatment supplies and personal protective equipment, and the fear of contracting the disease and infecting their families add to the problem.[36-37]. Nevertheless, COVID-19 has been ongoing for over two years, and the constant strain may exhaust healthcare workers' coping mechanisms.

Most of the socio-demographic and other variables assessed in this study were not statistically associated with burnout, reflecting the wide distribution of the problem among the participants despite such differences. We found that a lower number of household members, unmarried individuals, and physician jobs were the only variables associated with burnout among the participants. However, previous studies revealed inconsistent results on burnout risk factors among healthcare workers, and it is unclear whether that burnout follows a specific socio-demographic pattern among healthcare workers [29, 31, 35]. However, some of these associations with socio-demographic factors can be explainable. Among different medical professions, there are differences in the working duties, which put doctors at higher risk of violence and abuse. In our setting, doctors are the front-liners who deal with angry patients and their relatives, particularly when counseling them about their COVID-19 diagnosis and condition or when breaking the news that a patient has passed away [24, 37]. Furthermore, they are responsible for dealing with critically ill patients with a higher risk of complications and mortality [24].

Regarding the habits used to lessen burnout symptoms, seeking professional support to cope with moral distress and grief was significantly associated with lower rates of burnout among the participants. The participants have used other habits to alleviate the burnout symptoms, such as exercising, spiritual habits, and taking brief mental breaks throughout the day. However, none showed a significant association with burnout rates in this study.

The findings of this study acknowledge a high demand for appropriate support interventions and necessitate the search for more effective strategies to be implemented to reduce the risk of burnout among healthcare workers. Using different approaches to reduce burnout, such as training to improve stress-coping skills, can avoid or recover from emotional exhaustion and disengagement. In addition, managing the workplace and environment, improving systems, providing regular psychosocial support, and recruiting additional workers can help address organizational and workplace defects because hostile Working conditions contribute to stress and job dissatisfaction, resulting in depersonalization, emotional exhaustion, and lack of accomplishment [38,39].

Strengths and limitations

This is one of the few studies assessing the burnout burden among healthcare workers. The sample size of the participants, considered a low response population, is representative of generalizability to overall COVID-19 isolation centers in Khartoum, Sudan. The findings of this study need to be considered in the context of some limitations; as this is a cross-sectional study, it will be challenging to draw causative relationships. The self-reported nature of the study could raise the possibility of recall bias. Also, the study was done in five sites which might limit results generalization for all healthcare workers in all settings in the country and could compromise representativeness.

Conclusion

Our study has shown that nearly half of the healthcare workers in isolation centers in Khartoum have suffered from burnout during COVID-19. Several socio-demographic factors have contributed to the increased level of burnout, and multiple coping mechanisms have accounted for a lower level of burnout among healthcare workers. The findings of this study address the high demand for appropriate interventions to be implemented to reduce the risk of burnout among frontline healthcare workers.

Acknowledgment:

We want to thank Ahmed Abdalhalim Mohamed Omer, Omer Abdulmajed, Aya Zakareya Noor Hamid, Asmaa Mohamed Abbas, Aldoma Mohammed Adam Ismail, Elhadi elsiddig Elhadi, Mohammed AFEFE Hasan Salim, and Tagwa Badawe for their help in collecting the data and Mazin S. Haroun for his help in the research. Also, we would like to thank healthcare workers for participating in our study.

Contributors:

ESA, MMI, SSK, ROA, HMA, and ATA conceived and designed the study. EAH designed the statistical analysis plan, analyzed the data, and developed the figures and tables. ESA, MMI, SSK, ROA, HMA, ATA, EAH, and SOO reviewed and contributed to the interpretation. ESA, MMI, SSK, ROA, HMA, ATA, EAH, and SOO drafted the first version of the manuscript. KMA contributes to the study design and Supervision. All authors contributed to intellectual content during the drafting and revision of the work and approved the final version of the manuscript.

Funding:

The authors funded the research.

Data availability statement

The dataset generated during the current study is available upon request

Consent for publication:

All the authors gave their written and verbal consent for publication.

Conflict of interest:

The authors declare that they do not have any conflict of interest

References

1. Coronavirus disease (COVID-19) [Internet]. Who.int. [cited 2021 Dec 29]. Available from: https://www.who.int/health-topics/coronavirus
2. CDC. COVID-19 and your health [Internet]. Centers for Disease Control and Prevention. 2021 [cited 2021 Dec 29]. Available from: https://www.cdc.gov/coronavirus/2019-ncov/about/index.html
3. WHO declares public health emergency for novel Coronavirus [Internet]. Medscape. 2020 [cited 2021 Dec 29]. Available from: https://www.medscape.com/viewarticle/924596
4. Wee S-L, McNeil DG Jr, Hernández JC. W.H.o. Declares global emergency as Wuhan Coronavirus spreads. The New York Times [Internet]. 2020 30 January [cited 2021 29 December]. Available from: https://www.nytimes.com/2020/01/30/health/coronavirus-world-health-organization.html
5. Q5.U.S. to suspend most travel from Europe; N.b.a. pauses after player gets the virus. The New York Times [Internet]. 2020 Mar 11 [cited 2021 Dec 29]. Available from: https://www.nytimes.com/2020/03/11/world/coronavirus-news.html
6. SUDAN: Full lockdown in Khartoum. Africa Research Bulletin: Economic, Financial and Technical Series 2020 May;57. https://doi.org/10.1111/j.1467-6346.2020.09437.x
7. Kunna E. Sudan: Managing COVID-19 pandemic during a time of transition. Arab Reform Initiative [Internet]. 2020 [cited 2022 Jul 10]. Available from: https://www.arab-reform.net/publication/sudan-managing-covid-19-pandemic-during-a-time-of-transition/
8. Ahmed A, Mohamed NS, El-Sadig SM, Fahal LA, Abelrahim ZB, Ahmed ES, *et a*l. COVID-19 in Sudan. J Infect Dev Ctries. 2021 7 March;15:204-8. DOI: 10.3855/jidc.14520.
9. Sudan: WHO Coronavirus disease (COVID-19) dashboard with vaccination data [Internet]. Who.int. [cited 2022 Jul 10]. Available from: https://covid19.who.int/region/emro/country/sd
10. Coping with stress [Internet]. Cdc.gov. 2021 [cited 2021 Dec 29]. Available from: https://www.cdc.gov/mentalhealth/stress-coping/cope-with-stress/index.html
11. Burnout [Internet]. Mental Health UK. 2021 [cited 2021 Dec 29]. Available from: https://mentalhealth-uk.org/burnout/
12. Brooks SK, Webster RK, Smith LE, Woodland L, Wessely S, Greenberg N, *et al*. The psychological impact of quarantine and how to reduce it: a rapid review of the evidence. Lancet. 2020 Mar 14;395:912-20. DOI: 10.1016/S0140-6736(20)30460-8. Epub 2020 26 February.
13. Leo CG, Sabina S, Tumolo MR, Bodini A, Ponzini G, Sabato E, *et al*. Burnout Among Healthcare Workers in the COVID-19 Era: A Review of the Existing Literature. Front Public Health. 2021 29 October;9:750529. DOI: 10.3389/fpubh.2021.750529.
14. The Lancet. COVID-19: protecting healthcare workers. Lancet [Internet]. 2020 [cited 2021 Dec 29];395(10228):922. Available from: https://www.thelancet.com/journals/lancet/article/PIIS0140-6736(20)30644-9/fulltext
15. Søvold LE, Naslund JA, Kousoulis AA, Saxena S, Qoronfleh MW, Grobler C, *et al*. Prioritizing the mental health and well-being of healthcare workers: An urgent global public health priority. Front Public Health [Internet]. 2021 [cited 2021 Dec 29];9:679397. Available from: https://pubmed.ncbi.nlm.nih.gov/34026720/
16. Afulani PA, Gyamerah AO, Nutor JJ, Laar A, Aborigo RA, Malechi H, *et al*. Inadequate preparedness for response to COVID-19 is associated with stress and burnout among healthcare workers in Ghana. PLoS One. 2021 Apr 16;16:e0250294. DOI: 10.1371/journal.pone.0250294.

17. Patel RS, Bachu R, Adikey A, Malik M, Shah M. Factors related to physician burnout and its consequences: A review. Behav Sci (Basel) [Internet]. 2018;8:98. Available from: http://dx.doi.org/10.3390/bs8110098
18. Pélissier C, Charbotel B, Fassier JB, Fort E, Fontana L. Nurses' Occupational and Medical Risks Factors of Leaving the Profession in Nursing Homes. Int J Environ Res Public Health. 2018 Aug 27;15:1850. DOI: 10.3390/ijerph15091850.
19. Lin YY, Pan YA, Hsieh YL, Hsieh MH, Chuang YS, Hsu HY, *et al*. COVID-19 Pandemic Is Associated with an Adverse Impact on Burnout and Mood Disorder in Healthcare Professionals. Int J Environ Res Public Health. 2021 1 April;18:3654. DOI: 10.3390/ijerph18073654.
20. Nguyen J, Liu A, McKenney M, Liu H, Ang D, Elkbuli A. Impacts and challenges of the COVID-19 Pandemic on emergency medicine physicians in the United States. Am J Emerg Med. 2021 Oct;48:38-47. DOI: 10.1016/j.ajem.2021.03.088. Epub 2021 2 April.
21. Guastello AD, Brunson JC, Sambuco N, Dale LP, Tracy NA, Allen BR, Mathews CA. Predictors of professional burnout and fulfilment in a longitudinal analysis on nurses and healthcare workers in the COVID-19 pandemic. J Clin Nurs. 2022 Aug 10:10.1111/jocn.16463. doi: 10.1111/jocn.16463.
22. Gualano MR, Sinigaglia T, Lo Moro G, Rousset S, Cremona A, Bert F, Siliquini R. The Burden of Burnout among Healthcare Professionals of Intensive Care Units and Emergency Departments during the COVID-19 Pandemic: A Systematic Review. Int J Environ Res Public Health. 2021 Aug 2;18(15):8172. doi: 10.3390/ijerph18158172.
23. Brower KJ. Professional Stigma of Mental Health Issues: Physicians Are Both the Cause and Solution. Acad Med. 2021;96(5):635-40.
24. El-Sadig SM, Fahal LA, Abdelrahim ZB, Ahmed ES, Mohamed NS, Siddig EE. Impact of COVID-19 on doctors and healthcare providers during the pandemic in Sudan. Trans R Soc Trop Med Hyg. 2021;115(6):577-8.
25. Oldenburg Burnout Inventory (OLBI). (n.d.). MDApp. Retrieved 2 June, 2022, from https://www.mdapp.co/oldenburg-burnout-inventory-olbi-calculator-606/
26. Gruszczynska E, Basinska BA, Schaufeli WB. Within- and between-person factor structure of the Oldenburg Burnout Inventory: Analysis of a diary study using multilevel confirmatory factor analysis. PLOS ONE. 2021;16(5):e0251257.
27. Tipa RO, Tudose C, Pucarea VL. Measuring Burnout Among Psychiatric Residents Using the Oldenburg Burnout Inventory (OLBI) Instrument. J Med Life. 2019;12(4):354-60.
28. Summers RF, Gorrindo T, Hwang S, Aggarwal R, Guille C. Well-Being, Burnout, and Depression Among North American Psychiatrists: The State of Our Profession. Am J Psychiatry. 2020;177(10):955-64.
29. El Dabbah NA, Elhadi YAM. Burnout among frontline health professionals in two African countries: a cross-sectional study from Egypt and Sudan. 2021. doi: 10.21203/RS.3.RS-951355/V1
30. Elhadi YAM, Ahmed A, Salih EB, Abdelhamed OS, Ahmed MHH, El Dabbah NA. A cross-sectional survey of burnout in a sample of resident physicians in Sudan. PLOS ONE. 2022;17(3):e0265098.
31. Denning M, Goh ET, Tan B, Kanneganti A, Almonte M, Scott A, Martin G, Clarke J, Sounderajah V, et al. Determinants of burnout and other aspects of psychological well-being in healthcare workers during the Covid-19 Pandemic: A multinational cross-sectional study. PLoS One. 2021 Apr 16;16(4):e0238666. doi: 10.1371/journal.pone.0238666.

32. Elhadi M, Msherghi A, Elgzairi M, Alhashimi A, Bouhuwaish A, Biala M, Abuelmeda S, Khel S, Khaled A, Alsoufi A, Elmabrouk A, Alshiteewi FB, Hamed TB, Alhadi B, Alhaddad S, Elhadi A, Zaid A. Burnout Syndrome Among Hospital Healthcare Workers During the COVID-19 Pandemic and Civil War: A Cross-Sectional Study. Front Psychiatry. 2020 Dec 11;11:579563. doi: 10.3389/fpsyt.2020.579563.
33. Shoman Y, El May E, Marca S, Wild P, Bianchi R, Bugge M, et al. Predictors of occupational burnout: A systematic review. International Journal of Environmental Research and Public Health. 2021;18(17):9188
34. Sarboozi Hoseinabadi T, Kakhki S, Teimori G, Nayyeri S. Burnout and its influencing factors between frontline nurses and nurses from other wards during the outbreak of Coronavirus Disease -COVID-19- in Iran. Invest Educ Enferm. 2020 Jul;38(2):e3. DOI: 10.17533/udea.iee.v38n2e03.
35. Tan BYQ, Kanneganti A, Lim LJH, Tan M, Chua YX, Tan L, Sia CH, Denning M, Goh ET, Purkayastha S, Kinross J, Sim K, Chan YH, Ooi SBS. Burnout and Associated Factors Among Health Care Workers in Singapore During the COVID-19 Pandemic. J Am Med Dir Assoc. 2020 Dec;21(12):1751-1758.e5. doi: 10.1016/j.jamda.2020.09.035.
36. Elhadi M, Msherghi A, Elgzairi M, Alhashimi A, Bouhuwaish A, Biala M, Abuelmeda S, Khel S, Khaled A, Alsoufi A, Elhadi A, BenGhatnsh A. The Mental Well-Being of Frontline Physicians Working in Civil Wars Under Coronavirus Disease 2019 Pandemic Conditions. Front Psychiatry. 2021 Jan 14;11:598720. doi: 10.3389/fpsyt.2020.598720.
37. Elhadi YAM, Adebisi YA, Hassan KF, Mohammed SEE, Lin X, Lucero-Prisno Iii DE. The formidable task of fighting COVID-19 in Sudan. Pan Afr Med J. 2020 7 August;35(Suppl 2):137. doi: 10.11604/pamj.supp.2020.35.137.24984.
38. Pollock A, Campbell P, Cheyne J, Cowie J, Davis B, McCallum J, et al. Interventions to support the resilience and Mental Health of Frontline Health and social care professionals during and after a disease outbreak, epidemic or pandemic: A mixed methods systematic review. Cochrane Database of Systematic Reviews. 2020;2020(11).
39. Mengist B, Amha H, Ayenew T, Gedfew M, Akalu TY, Assemie MA, et al. Occupational stress and burnout among health care workers in Ethiopia: A systematic review and meta-analysis. Archives of Rehabilitation Research and Clinical Translation. 2021;3(2):100125.

27-janouary-2023
Author's response to reviews
Subject: Revision and resubmission of PONE-D-22-28346

Dear editor,
On behalf of all the contributing authors, I would like to express our sincere appreciation of the reviewers' constructive comments concerning our article entitled "Burnout and its associated factors among healthcare workers in Khartoum's COVID-19 isolation centers: A cross-sectional study"

We have revised our manuscript according to the reviewers' comments, questions, and suggestions. We believe that the manuscript has been further improved and the revised paragraphs were highlighted in yellow in the manuscript. If there are any other modifications we could make, we would very much like to modify them and we appreciate your help. Below are point-by point responses to the reviewers' comments.

Your sincerely

Reviewer 1:
The manuscript, although it gets the meaning across, is not very well written. It should be re-written to bring the standard close to that of the Journal's. The Discussion Section in particular can be improved; although studies have been included but their findings have not been connected with those of the study being discussed.
response to reviewer 1:
Thank you for your comment. The manuscript was revised and corrected, and the discussion section was improved according to your note.

Reviewer 2:
Comment: In abstract you should show prevalence of burnout
Response: done

Comment: -There should not be any abbreviations in the abstract.
Response: all abbreviations in the abstract were removed

Comment: -In introduction you should give a definition of job burnout
Response: a brief description of burnout was added (end of page 4)

Comment: -You should describe more literature on their association and on their prevalence in healthcare professionals during the covid 19 pandemic.
Response: more description was added to the introduction section (end of page 4)

Comment: -The discussion needs significant reworking. It does not explicate the reasons for nurses to have burnout.
Response: discussion section was revised and edited according to your note. We think the reviewer meant 'doctors' not nurses in his comment (see table 4)

Comment: Many bibliographies are obsolete. The bibliographic citations used are more than 5 years old. The authors must update and arrange the bibliography. Some references are incomplete or have errors. The authors should review this section

Response: we re-checked all references to ensure that all of them are not older than 2018
