# Supplementary material for: Burnout and its associated factors among healthcare workers in COVID-19 isolation centres in Khartoum, Sudan: A cross-sectional study
Source: PLoS One. 2023 Jul 21;18(7):e0288638. doi: 10.1371/journal.pone.0288638 (PMC10361487; doi:10.1371/journal.pone.0288638)
Supplement: Attachment — Submitted filename: Response to Reviewers.docx [file pone.0288638.s003.docx]

**27-janouary-2023**

**Author’s response to reviews**

**Subject: Revision and resubmission of PONE-D-22-28346**

Dear editor,

On behalf of all the contributing authors, I would like to express our sincere appreciation of the reviewers’ constructive comments concerning our article entitled “Burnout and its associated factors among healthcare workers in Khartoum's COVID-19 isolation centers: A cross-sectional study”

We have revised our manuscript according to the reviewers’ comments, questions, and suggestions. We believe that the manuscript has been further improved and the revised paragraphs were highlighted in yellow in the manuscript. If there are any other modifications we could make, we would very much like to modify them and we appreciate your help. Below are point-by point responses to the reviewers' comments.

Your sincerely

Reviewer 1:

The manuscript, although it gets the meaning across, is not very well written. It should be re-written to bring the standard close to that of the Journal's. The Discussion Section in particular can be improved; although studies have been included but their findings have not been connected with those of the study being discussed.

**response to reviewer 1:**

**Thank you for your comment. The manuscript was revised and corrected, and the discussion section was improved according to your note.**

Reviewer 2:

Comment: In abstract you should show prevalence of burnout

**Response: done**

Comment: -There should not be any abbreviations in the abstract.

**Response: all abbreviations in the abstract were removed**

Comment: -In introduction you should give a definition of job burnout

**Response: a brief description of burnout was added (end of page 4)**

Comment: -You should describe more literature on their association and on their prevalence in healthcare professionals during the covid 19 pandemic.

**Response: more description was added to the introduction section (end of page 4)**

Comment: -The discussion needs significant reworking. It does not explicate the reasons for nurses to have burnout.

**Response: discussion section was revised and edited according to your note. We think the reviewer meant ‘doctors’ not nurses in his comment (see table 4)**

Comment: Many bibliographies are obsolete. The bibliographic citations used are more than 5 years old. The authors must update and arrange the bibliography. Some references are incomplete or have errors. The authors should review this section

**Response: we re-checked all references to ensure that all of them are not older than 2018**
